# Structural Basis for the Aminoacid Composition of Proteins from Halophilic Archea

**DOI:** 10.1371/journal.pbio.1000257

**Published:** 2009-12-15

**Authors:** Xavier Tadeo, Blanca López-Méndez, Tamara Trigueros, Ana Laín, David Castaño, Oscar Millet

**Affiliations:** 1 Structural Biology Unit, CIC bioGUNE, Derio, Spain; 2 Institute of Research in Biomedicine, Parc Científic de Barcelona, Barcelona, Spain; Brandeis University, United States of America

## Abstract

Proteins from halophilic organisms, which live in extreme saline conditions, have evolved to remain folded at very high ionic strengths. The surfaces of halophilic proteins show a biased amino acid composition with a high prevalence of aspartic and glutamic acids, a low frequency of lysine, and a high occurrence of amino acids with a low hydrophobic character. Using extensive mutational studies on the protein surfaces, we show that it is possible to decrease the salt dependence of a typical halophilic protein to the level of a mesophilic form and engineer a protein from a mesophilic organism into an obligate halophilic form. NMR studies demonstrate complete preservation of the three-dimensional structure of extreme mutants and confirm that salt dependency is conferred exclusively by surface residues. In spite of the statistically established fact that most halophilic proteins are strongly acidic, analysis of a very large number of mutants showed that the effect of salt on protein stability is largely independent of the total protein charge. Conversely, we quantitatively demonstrate that halophilicity is directly related to a decrease in the accessible surface area.

## Abstract 2

In order to survive in highly saline environments, proteins from halophilic archea have evolved with biased amino acid compositions that have the capacity to reduce contacts with the solvent.

## Author Summary

Life on earth exhibits an enormous adaptive capacity and living organisms can be found even in extreme environments. The halophilic archea are a group of microorganisms that grow best in highly salted lakes (with KCl concentrations between 2 and 6 molar). To avoid osmotic shock, halophilic archea have the same ionic strength inside their cells as outside. All their macromolecules, including the proteins, have therefore adapted to remain folded and functional under such ionic strength conditions. As a result, the amino acid composition of proteins adapted to a hypersaline environment is very characteristic: they have an abundance of negatively charged residues combined with a low frequency of lysines. In this study, we have investigated the relationship between this biased amino-acid composition and protein stability. Three model proteins – one from a strict halophile, its homolog from a mesophile and a totally unrelated protein from a mesophile - have been largely redesigned by site-directed mutagenesis, and the resulting mutants have been characterized structurally and thermodynamically. Our results show that amino acids with short side-chains (like aspartic and glutamic acid) are preferred to the longer lysine because they succeed in reducing the interaction surface between the protein and the solvent, which is beneficial in an environment where water is in limited availability because it also has to hydrate the salt ions.

## Introduction

Halophilic archea are extremophiles that thrive in highly saline environments such as natural salt lakes [1]. To maintain positive turgor pressure, salt concentration in the cytoplasm can reach 4 M [2]. Proteins from these organisms have evolved to maximize stability and activity at high salt concentrations (haloadaptation) [3],[4]. Comparative analyses between the proteomes of halophilic and non-halophilic bacteria have recognized a characteristic signature in the amino acid composition of proteins with hypersaline adaptation [5],[6]. These features include a large increase in glutamic acids and, more frequently, aspartic acids; a drastic drop in the number of lysines (often replaced by arginines) [7]; and a decrease in the overall hydrophobic content [5],[8]. The same trends are observed in taxonomically distant species, and convergent evolution to a unique solution for halophilic adaptation suggests that the same fundamental mechanism is operating [5]. Understanding the haloadaptation mechanism is of particular interest given the influence of salt on function, folding, oligomerization, and solubility, and has obvious potential application in the biotechnological industry. Structural comparison of related halophilic and mesophilic proteins has revealed that changes are concentrated at the protein surface [6],[9]–[12]. It has been suggested that haloadaptation and salt modulation of the hydrophobic effect have a common origin [13]. An alternative hypothesis suggests that hydrated ions can interact with surface acidic residues (a.r.) to stabilize the folded conformation [14],[15].

Here, we have investigated the mechanism of hypersaline adaptation in protein stability by extensive site-directed mutagenesis followed by a thermodynamic and structural characterization of the protein derivatives using three different domains: the halophilic 1A domain of the NAD^+^-dependent DNA ligase N (*Hv* 1ALigN) from *Haloferax volcanii* [16], the homologous domain from E.coli (*Ec* 1ALigN), and the mesophilic IgG binding domain of the protein L from *Streptococcus magnus* (ProtL) [17]. *Hv* 1ALigN and *Ec* 1ALigN are functionally identical and have a 30% sequence homology, whereas ProtL and 1ALigN are not structurally nor functionally related. The three domains unfold reversibly according to a two state model. The wild type of *Hv* 1ALigN requires potassium chloride to fold and forms part of an enzyme with optimal activity at 3.2M KCl [16]. The wild types *Ec* 1ALigN and ProtL show no changes in stability upon addition of sodium or potassium chloride [18]. Our results show that net charge exerts a negligible effect on the protein hypersaline adaptation mechanism. Conversely, the abundance of aspartate and glutamate residues results in a decrease in the protein's solvent-accessible area, which is the key mechanism for haloadaptation. By altering the amino acid composition on the protein surface, it is possible to modify the salt dependence of protein stability and interconvert halophilic and mesophilic proteins.

## Results

### Effect of Salt on the Stability of Wild Type *Hv* 1ALigN, *Ec* 1ALigN, and ProtL

In the presence of 1 M KCl, *Hv* 1ALigN has a circular dichroism (CD) spectrum typical for a folded protein with a significant degree of helical content and very similar when compared to the *Ec* 1ALigN CD spectrum, indicating that the two proteins are structurally homologous (see Figure S1). In the absence of salt, *Hv* 1ALigN partially unfolds. At KCl concentrations below 0.5M, *Hv* 1ALigN shows a strong non-linear stabilization with the cosolute and it becomes fully folded at concentrations higher than 0.35 M (see Figure S2). ProtL, *Hv* 1ALigN, and *Ec* 1ALigN show a fully reversible thermal and chemical unfolding. Temperature-, urea-, or guanidinium chloride-induced denaturation curves monitored by CD and fluorescence spectroscopy were used to measure the changes in stability induced by NaCl or KCl (Δ*G_salt_*, see Protocol S1 for details). The free energy at 3.2 M KCl (or NaCl) has been determined to measure the haloadaptation of the proteins. Moreover, upon salt addition, their stabilities are proportional to the molar concentration of the cosolute, and the slope (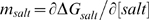) was also used to quantify the protein's hypersaline adaptation. Wild type ProtL and *Ec* 1ALigN stabilities remain unaffected by the presence of KCl or NaCl (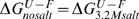 and *m_KCl_* = *m_NaCl_* = 0 kcal·mol^−1^·M^−1^, Figures S3 and S4) [18]. At concentrations higher than 0.5 M, the *Hv* 1ALigN domain shows a linear increase in stability upon KCl addition (slope *m_KCl_* = 0.30±0.05 kcal·mol^−1^·M^−1^, Figure S4).

### Mutation Design

Given the skewed amino acid composition of halophilic proteins, we performed a systematic mutational study involving the following surface amino acids: aspartic acid, glutamic acid, lysine, arginine, asparagine, serine, and glutamine. Mutations were grouped in three classes: (i) charge-preserving mutations that modify side chain length: E to D, DN to EQ, K to R, and R to K (XY to WZ involves a change from X or Y to W or Z, respectively); (ii) size preserving mutations that change the protein's charge: NQ to DE and DE to NQ; and (iii) mutations that change both size and charge: K to QES and DES to K. A cumulative strategy was used to amplify the response by introducing multiple mutations of the same type (XYx*n*WZ represents a mutant with *n* substitutions of this specific class). The complete list of 102 mutants, including the specific positions of the residues involved, is shown in the supplementary materials (Tables S1, S2, and S3).

**Figure 1:**
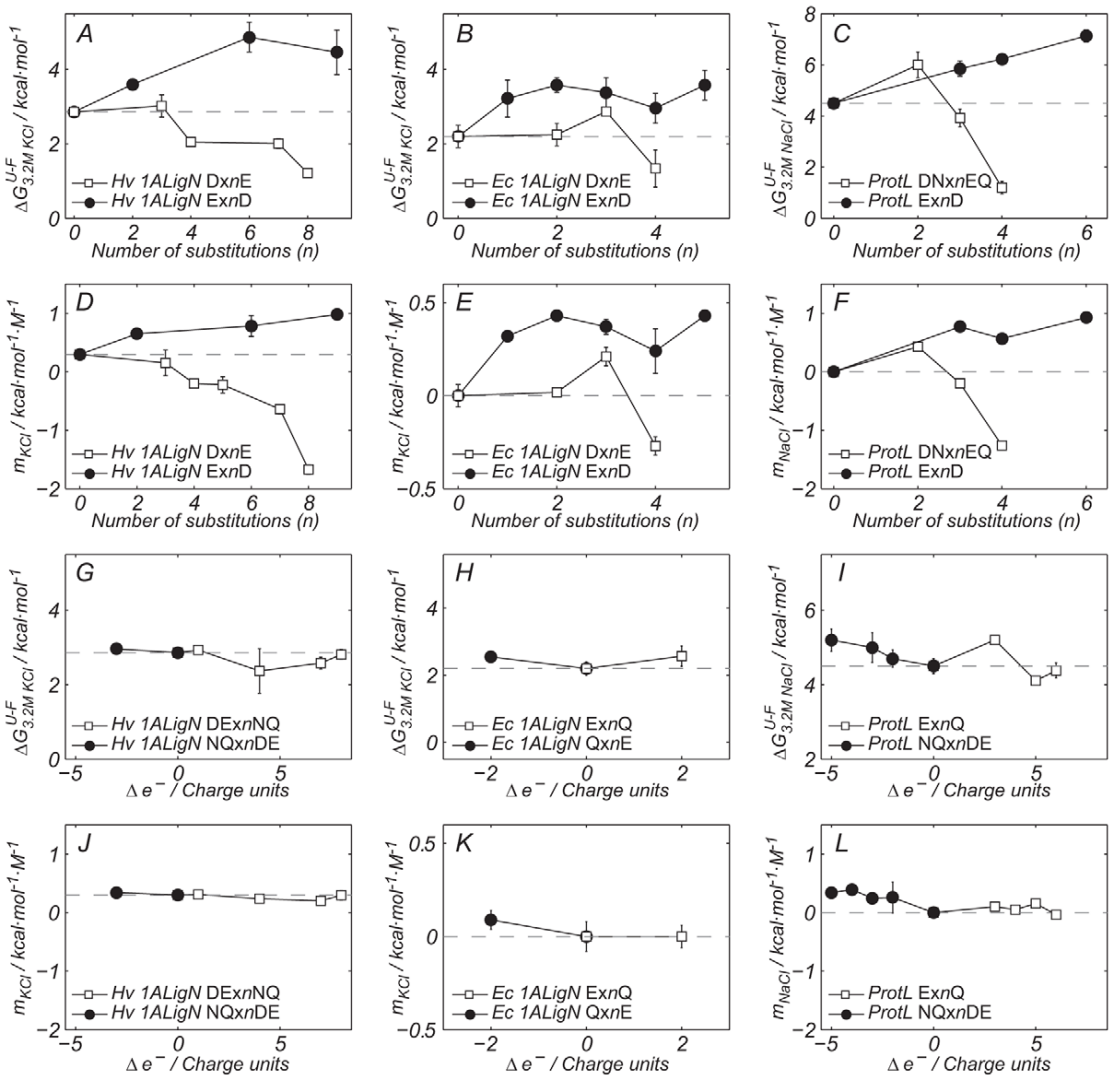
The effect of charge and side chain length in protein haloadaptation. The effect of the chain size (panels A to F) and charge (panels G to L) in haloadaptation of ProtL, *Hv* 1ALigN, and *Ec* 1ALigN was investigated by measuring the free energy at 3.2 M salt (KCl or NaCl) versus the number of substituted residues in the multiple mutations (*n* in XYx*n*WZ). In addition, the variation in protein stability induced by salt (*m_KCl_* or *m_NaCl_*) is also reported. For each panel, proteins and mutation classes are specified in the enclosed legend. Δe^−^ is defined as the residual theoretical charge upon mutation (mutant minus wild type). Error bars result from propagation of the experimental uncertainties in the *T_m_* values, by Montecarlo analysis. Dashed lines represent the 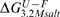 or the *m_salt_* values for wild type proteins. A negative *m_salt_* value means that the cosolute destabilizes the protein.

**Figure 2:**
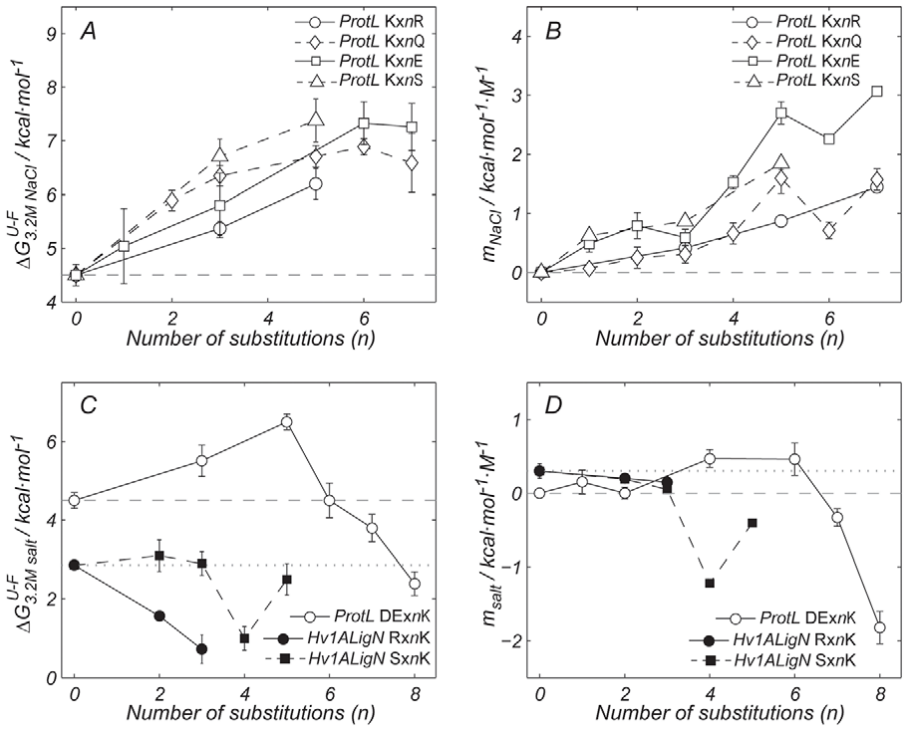
The low prevalence of lysines in the amino acid composition of halophilic proteins. The effect of lysines on protein haloadaptation was investigated by measuring the change in stability induced by salt (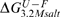, *m_KCl_*, and *m_NaCl_*) versus the number of substituted residues in the lysine involving mutations (*n* in XYx*n*WZ). For each panel, proteins and mutation classes are specified in the enclosed legend. Error bars result from propagation of the experimental uncertainties in the *T_m_* values, by Montecarlo analysis. Dashed and dotted lines represent the 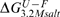 or the *m_NaCl_* and *m_KCl_* for wild type ProtL, *Hv* 1A LigN, and *Ec* 1ALigN. A negative *m_salt_* value means that the cosolute destabilizes the protein.

To evaluate the effect of the surface modifications in hypersaline adaptation, the free energies at 3.2 M salt (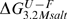) have been determined by equilibrium denaturation experiments and are plot versus the number of substituted residues in Figures 1 and 2. Moreover, the protein's mid-denaturation point (*T_m_*) varies linearly with the molar concentration of NaCl or KCl (see Figures S3 and S4), and the experimental *m_salt_* values are also plotted in Figures 1 and 2. Panels A to F (G to L) in Figure 1 show the effect of the mutants that conserve the charge (size). Reduction in the side chain by one methylene without altering overall charge (E for D) improves the halophilic adaptation of *Hv* 1ALigN (black circles in panels A and D) and generates a salt-dependent stabilizing effect in the mesophilic proteins *Ec* 1ALigN (black circles in panels B and E) and ProtL (black circles in panels C and F). Changing aspartate to bulkier glutamate has the opposite effect and *Hv* 1ALigN mutants are not only less stabilized by salt than the wild type, but when four or more aspartates are replaced by glutamates, potassium chloride *destabilizes Hv* 1ALigN (white squares in panels A and D). A similar effect is observed for *Ec* 1ALigN and ProtL, and DNx4EQ is strongly destabilized by NaCl (white squares in Figures 1C and 1F). These results provide an explanation for the preference for aspartate over glutamate in halophilic proteins.

Halophilic proteins typically have very low isoelectric points. Substitution of all the N and Q residues to D and E would cause an increase of 15% in the theoretical negative charge for *Hv* 1ALigN. In the case of ProtL the density of a.r. would change from 2.23 to 3.35 a.r./10^3^Å^2^ (the average value for halophiles is 4.07 a.r./10^3^ Å^2^) [9]. As shown in Figures 1G and 1J (black circles), within experimental error no changes in the 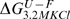 or the *m_KCl_* values were observed for this type of mutant in *Hv* 1ALigN and only a minor increase in the halophilic character (higher stability in the presence of salt) was found in ProtL (black circles in Figures 1I and 1L). The same result is found when two Q residues are replaced by E in *Ec* 1ALigN (Figures 1H and 1K, black circles). Negative-charge reducing mutations were also studied (see white squares in Figures 1G to 1L) and none of the three proteins changed their salt stabilization by a reduction in the a.r. content. Thus, charge variation has little effect on the modulation of the halophilicity of *Hv* 1ALigN, *Ec* 1ALigN, and ProtL in the explored Δe^−^ range, in contrast to the large effects observed after altering the size of the side chains.

A low occurrence of lysines combined with a slight increase in the arginine content is another feature of the amino acid composition of halophilic proteins [5]. ProtL has seven lysines out of 64 residues, which is about the average percentage for mesophilic proteins. As shown in Figures 2A and 2B, changing K for R leads to salt-dependent stabilization with a magnitude of 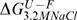 (or *m_NaCl_*) almost proportional to the number of replaced lysines. A similar result is obtained when K is replaced by other polar residues with smaller side chains Q, S, or E, independently of their charge. On the other hand, ProtL becomes destabilized by NaCl if a sufficient number of lysines are introduced in place of a.r. (white circles in Figures 2C and 2D). Finally, when three lysines are incorporated into the surface of *Hv* 1ALigN, a decrease in the protein stability at high salt concentration is observed (black circles in Figure 2C). When lysines are incorporated in place of serines (black squares in Figure 2C, 2D), this trend is also clear and *m_KCl_* becomes negative. Thus, the low prevalence of K in proteins from halophilic archea can be explained by the destabilizing effect, at high salt concentrations, of the long lysine side chain.

The *m_salt_* and 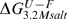 measurements should differ due to the fact that only the latter takes into account the intrinsic effect of the mutation in protein stability. The experimental 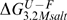 values are compared to the predicted values obtained from the *m_salt_* slopes in Figure S5. A reasonable correlation is found and, in general, small changes in stability are introduced with the mutation when compared with the effect of salt in stability, consistent with the conservative mutational design. Outliers in this correlation correspond to the mutants that produce larger changes in protein stability upon mutation. For this subset of mutants, an increase in protein stability is found by raising the ionic strength up to 0.25 M KCl (unpublished data), suggesting an electrostatic origin for their destabilization.

Surface modifications with alternative mutation pathways have also been constructed and tested for six different mutation designs (data shown in Figure S6). For this limited dataset, the different positional mutants show equivalent trends with respect to *m_salt_*, highlighting the importance of the number of surface mutations of a given type in the modulation of *m_KCl_* and *m_NaCl_*. This is in contrast to the effect of the mutation on the intrinsic stability in the absence of cosolute, which is determined by both the nature and the specific location of the mutation.

### Changes in Solubility upon Mutation

Surface mutations can significantly affect the solubility properties of the protein. We have determined the solubility for wild type ProtL and two mutants: Kx4Q and Kx4E. As shown in Table 1, four new glutamines produce no changes in the soluble fraction while a dramatic increase in solubility is observed upon introduction of four glutamates. Thus, the large number of Asp and Glu residues found in the surface of halophilic proteins enhances the solubility of the protein in an environment of reduced water activity.

**Table 1:** Solubility of ProtL variants in 20 mM phosphate, 2M ammonium sulphate at pH 6.0, and 25°C.

ProtL Variant	Solubility (mg/mL)
w.t.	50±8
Kx4Q	56±7
Kx4E	>85±8^a^

a At this concentration no precipitation could be observed.

### Correlation between Haloadaptation and the Reduction in Surface Area

**Figure 3:**
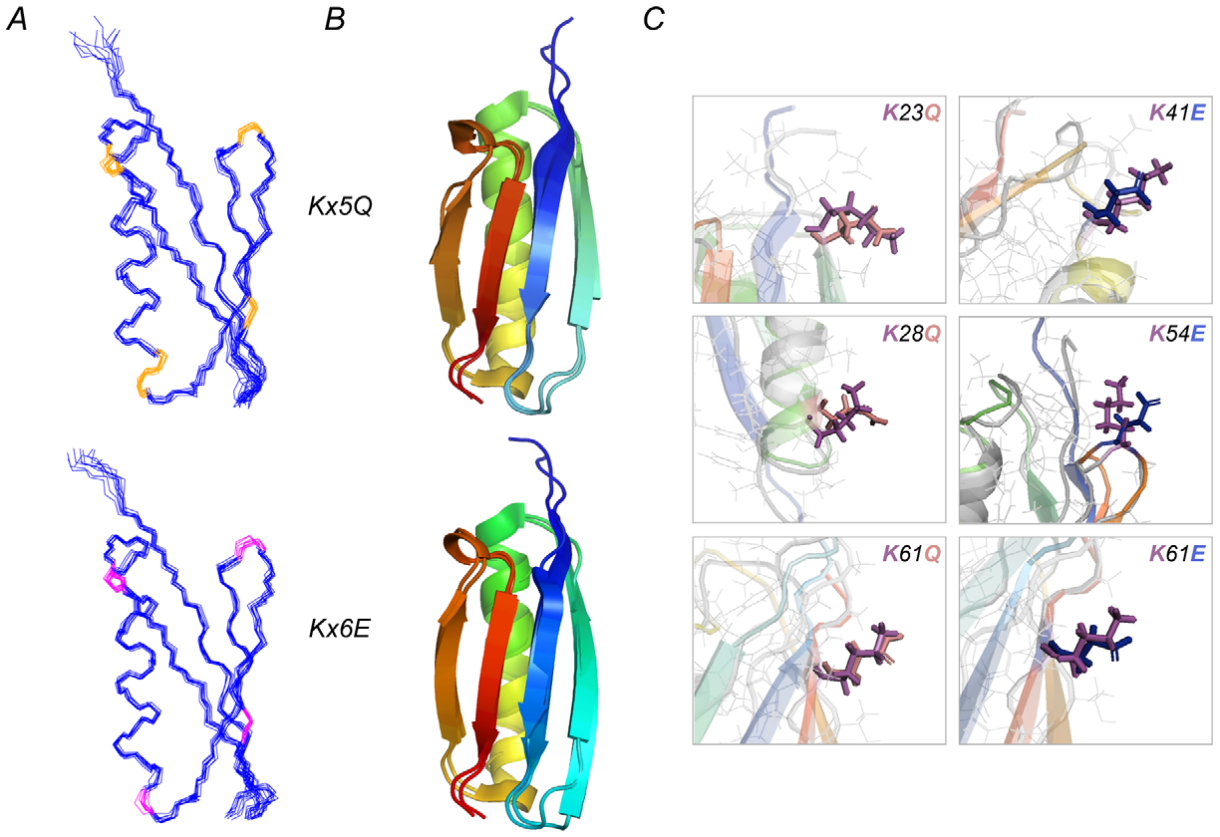
Structural characterization of the KxnQE mutants. Using NMR spectroscopy, the high-resolution structures of ProtL Kx5Q and ProtL Kx6E were obtained. The 10 lowest energy-refined conformers are shown in (A). The mutated residues are highlighted in yellow or red for K to Q and K to E mutations, respectively. The alignment between the mutant structures and wild type ProtL (1hz6) [19], shown in (B), reveals that the changes introduced upon mutation are minimal. The spatial distribution of the mutated side chains is generally very similar to the wild type. (C) shows close-up views for a representative selection of wild type and mutant side chains that have been aligned. Lysine side chains are colored in magenta whereas glutamine and glutamate side chains are colored in pink and blue, respectively.

To quantify the effect of mutations involving changes in surface area we determined high resolution NMR structures of two multiple mutants of ProtL: Kx5Q ProtL and Kx6E ProtL (Figure 3A, PDB accession codes 2jzp and 2kac, respectively). Kx6E is an obligate halophile and the structure was solved in the presence of 500 mM NaCl. The structures of the mutants are very similar to that of wild type ProtL (1hz6) [19] with root mean square deviation values of 0.58 Å and 0.69 Å for Kx5Q and Kx6E, respectively (Figure 3B and Table S4). The overall folding and the structure of the core are totally conserved and changes are localized on the protein surface. No new inter-side-chain interactions were observed in the mutant structures and the mutated side chains are placed in a very similar conformation when compared to the wild type (the overlap between the solution wild type structure and some representative side chains of Kx5Q or Kx6E is shown in Figure 3C).

**Figure 4:**
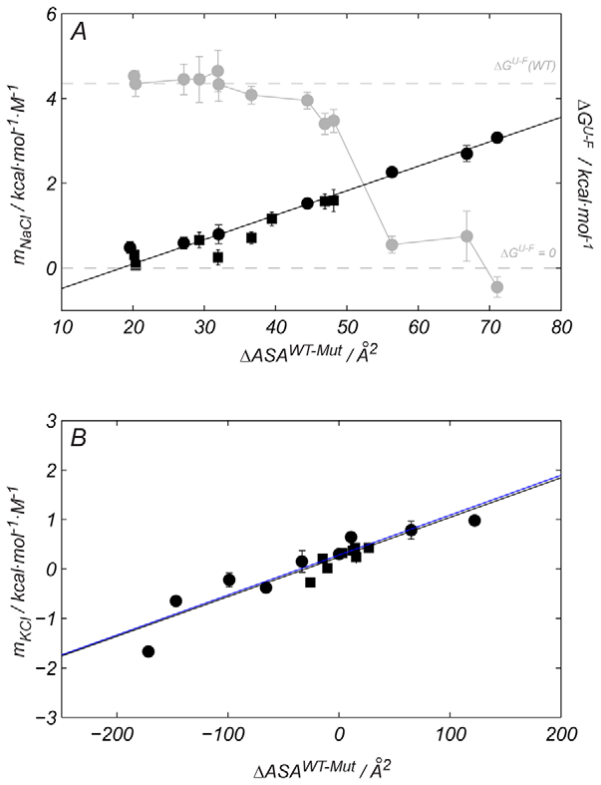
A metric for ProtL haloadaptation. (A) The mesophilic ProtL can be converted from a folded protein that does not become stabilized by salt (Δ*G^U−F^*>0, *m_NaCl_* = 0, wild type) to an obligate halophile (Δ*G^U−F^*<0, *m_NaCl_*>0, Kx7E). This transition is a continuous process as demonstrated by the empirical correlation found between the *m_NaCl_* and Δ*ASA^WT−Mut^* for the ProtL mutants: K for Q (black squares) and K for E (black circles). This data plot uses the left ordinate axis for the *m_NaCl_* units. (B) An equivalent correlation can also be found for the D for E and E for D mutants *Hv* 1ALigN (black circles) and *Ec* 1ALigN (black squares). Error bars result from propagation of the experimental uncertainties in the *T_m_* values, by Montecarlo analysis. The solid black line corresponds to the best linear fit of the data. In (B), black line corresponds to the linear fit including *Hv* and *Ec* 1ALigN whereas the fit of *Hv* 1ALigN is depicted with a blue line. For each protein, the stability upon unfolding at 298 K is shown in gray circles (units on the right ordinate axis). Error bars reflect the mean value from all the experimental measurements. Dashed gray lines highlight the Δ*G^U−F^* values for wild type ProtL (Δ*G^U−F^* (WT)) and when Δ*G^U−F^* = 0, as indicated.

Structural data were used to calculate the changes in the accessible solvent area in the folded conformation upon mutation (Δ*ASA^WT−Mut^*). As shown in Figure 4A, the increase in the halophilic character experienced by the mutants (encoded in the magnitude of *m_NaCl_*) shows a good correlation with the changes in the side chain's accessible solvent area introduced upon mutation: a reduction in the solvent-exposed area leads to an increase in *m_NaCl_*. Moreover, *m_NaCl_* is uncorrelated with Δ*e* ^−^ introduced upon mutation, underlining the low influence that the residue's charge has on the mechanism of haloadaptation for ProtL. The surface changes for the D to E and E to D mutants of *Hv* 1ALigN and *Ec* 1ALigN (shown in Figure 1A) have also been estimated. Since there are no high resolution structures for *Hv* 1ALigN, homology models have been used to calculate the changes in solvent accessible area upon mutation. As shown in Figure 4B, the *m_KCl_* variation is also correlated with the changes in area introduced upon mutation. These correlations establish a direct link between a metric for haloadaptation and a structural protein feature.

**Figure 5:**
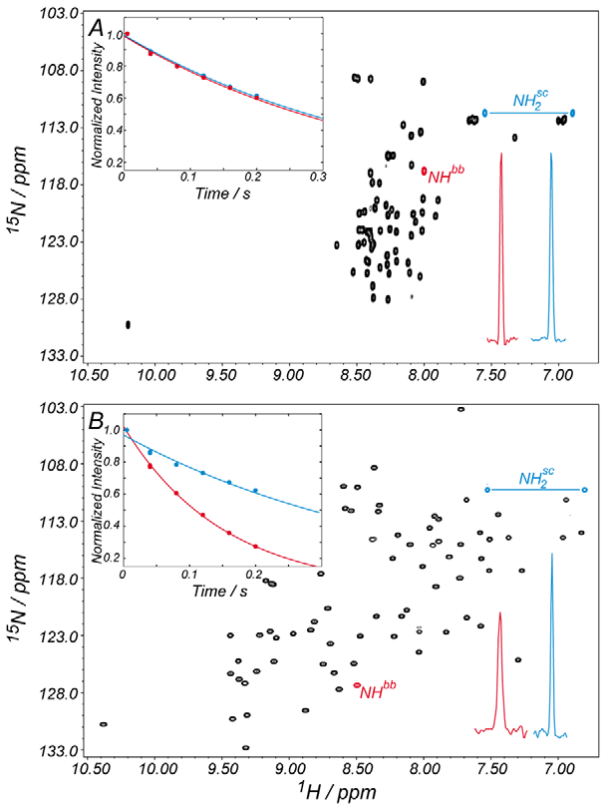
Salt-dependent folding of ProtL Kx7E. In the absence of salt the ^1^H,^15^N-HSQC spectrum for ProtL Kx7E shows a peak pattern typical of an unfolded protein, with a small peak dispersion in the proton dimension (A). The similarity in the transversal relaxation times (*T*_2_(^15^ *N*)) for the main chain (*NH^bb^*, red) and the side chain (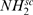, blue) nitrogen nuclei (inset of panel A) indicates fast tumbling of the polypeptide chain, a feature of unfolded proteins. Upon addition of salt (2 M NaCl), the peak pattern in the spectrum expands (B) and the *T*_2_(^15^ *N*) of the main chain and the side chains can be discriminated (inset of panel B), as expected for a folded protein of this size. The same trend is manifested in the peak intensities of the HSQC spectra and the traces for the considered peaks are also shown in the bottom right-hand corners of the spectra. Solid lines in the figure insets correspond to the best exponential fitting to the experimental data (filled circles).

The stability of the different ProtL mutants in the absence of salt at 298 K was determined by chemical denaturation experiments (gray circles in Figure 4A). Reduction of the solvent accessible area introduced upon mutation causes a progressive destabilization of the molecule, probably due to a reduction in the protein's hydrophobic effect. As a consequence, mutations increasing salt-induced stabilization also destabilize the protein in the absence of salt, converting a mesophilic protein into an obligate halophilic form, a trend found in natural halophilic proteins. An example is Kx7E ProtL, which is unfolded at 298 K in the absence of salt since ΔG^U−F^(Kx7E)<0. The ^1^H,^15^N-HSQC spectrum of ProtL Kx7E (in 20 mM sodium phosphate) shows a peak dispersion that is characteristic for an unfolded protein (Figure 5A). Protein dynamics, encoded in the transversal relaxation times, also agree well with an unfolded, highly flexible state. This mutant has the largest *m_NaCl_* indicating that very large stabilization with NaCl is expected. Consistent with this, in the presence of 2 M NaCl the ^1^H,^15^N-HSQC spectrum changes to a spectrum characteristic of a folded protein (Figure 5B), sharing a very similar peak pattern with wild type ProtL (Figure S7). Moreover, in 2 M NaCl, the relaxation times for the backbone nitrogen nuclei are much shorter than the side chain ones, as expected for a folded protein of this size.

## Discussion

The halophilic *Hv* 1ALigN domain is stabilized by salt while the stability of the mesophilic *Ec* 1ALigN and ProtL domains are completely independent of ionic strength. The *Hv* 1ALigN non-linear stabilization at [KCl]<0.5 M can be attributed to electrostatic effects whereas the linear dependence with salt found at [KCl]>0.5 M shall obey to a different mechanism. Surface residue replacements in the three proteins can increase or decrease the salt-induced stabilization or can even make it a destabilizing effect, depending on the nature and the number of surface residue replacements. Mutations leading towards the amino acid composition found in halophilic organisms increase the salt-induced stabilization, quantified by 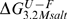 and, to a lesser extent, by *m_salt_*, the slope of the ΔG versus salt concentration plot.

Here we show that small side chains contribute to enhancement of salt-induced protein stability, and that the excellent correlations between *m_salt_* and Δ*ASA^WT−Mut^* underlie the role that surface packing may play in this mechanism. Proteins with a decreased solvent accessible surface are preferred in an environment where water molecules also have to solvate the ions. The stability of a halophilic protein at high salt concentrations will result from the balance of the side chain contributions to m*_salt_* and to the stability in the absence of cosolute. For the Kx*n*E mutation design in ProtL, chemical denaturation experiments revealed that the introduction of a.r. also causes protein destabilization if NaCl is not present, offering a plausible explanation for the large number of obligate halophilic proteins (that unfold in the absence of salt) found in nature. However, this is not the case for the majority of mutants studied in the present work where minor effects in stability have been found in the absence of salt, regardless of the nature or the number of substitutions. Examples of massive surface mutations into negative charged residues with minimal effects in stability have also been reported in the literature [20].

As shown in Figure 1 altering the number of negative charges on the protein surface results in almost no variation in stability modulation by salt indicating that D and N should be equally found in the aminoacid composition of halophilic proteins. Why are then (charged) aspartate/glutamate preferred over (neutral) asparagine/glutamine? D and E are among the hydrophilic residues that most contribute to increase protein solubility [21]. Our solubility measurements with ProtL are consistent with this idea. By accumulating a large number of negative charged residues in the surface, the tendency to aggregation will be reduced and the solubility at physiological pH will increase due to the lowering in the isoelectric point.

A decrease in the number of lysine residues results in an increase in halophilicity, independently of the charge, suggesting that the effect arises from the removal of the long lysine side chain. Inspection of Figure 2 reveals that substitution of lysine by glutamate indeed causes an increase in 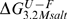 and *m_salt_* (white squares), consistent with the amino acid composition found in halophilic proteins. Moreover, structures of Kx5Q and Kx6E show that the replacement of lysine by glutamate results in a higher reduction of the solvent-exposed surface area than the substitution of glutamine.

Depending on the natural environment, hypersaline adaptation involves KCl or NaCl. However, our data are similar for both salts, indicating that a common mechanism prevails. The linear dependence of stability with the molar concentration of the salt suggests the existence of weak non-specific interactions [22],[23]. Some preliminary data with anions other than chloride indicate a cosolute-dependent modulation of protein stability following the Hofmeister series. Thus, salt-induced stabilization can probably be described in terms of the preferential interaction and preferential exclusion of the ions from the protein surface.

## Materials and Methods

### Cloning, Site-Directed Mutagenesis, and Protein Sample Preparation

The *Hv* 1ALigN and *Ec* 1ALigN were amplified by PCR reaction using the forward and reverse primers: 5′-GGGGACAAGTTTGTACAAAAAAGCAGGCTTCCCTCCGACCGAGTTCGAA-3′ and 5′-GGGGACCACTTTGTACAAGAAAGCTGGGTCCTAGACGTGTTCGACCGTCTC-3′ for *Hv* 1ALigN and 5′-GGGGACAAGTTTGTACAAAAAAGCAGGCTTCATGGAATCAATCGAACAACAACT-3′ and 5′-GGGGACCACTTTGTACAAGAAAGCTGGGTCCTAGAAAGCCGCCAGCGGCGC-3′ for *Ec* 1ALigN and subsequently cloned into a pDEST17 expression vector using the Gateway Technology (Invitrogen). The clones have been deposited in the addGENE plasmid bank (ID: 20024 and 22296 for *Hv* LigN and *Ec* LigN, respectively). Site-directed mutagenesis was performed using the commercial QuiaChange II Kit (Quiagen) with custom made oligonucleotides as PCR reaction primers (Invitrogen). The primers used to obtain the 102 studied mutants are listed in the supplementary materials. Freshly transformed *Escherichia coli* BL21 DE(3) and BL21 AI cells were used for ProtL and *Hv* or *Ec* 1ALigN protein expression and were induced with isopropyl-beta-D-thiogalactopyranoside (IPTG) and L-arabinose, respectively. Samples were grown in the appropriate media depending on the experiment: LB rich media for the CD, fluorescence, and calorimetric experiments and M9 minimal media with the appropriate isotope labeling for the NMR experiments. ProtL purification was achieved by a thermal shock followed by gel filtration chromatography (Superdex 75, GE Healthcare) and buffer exchange to 20 mM phosphate buffer at pH 6.0. For *Ec* 1ALigN, cell pellets were resuspended in 6 M guanidinium chloride and protein was refolded by fast dilution into 20 mM phosphate buffer, at pH 8.0, followed by gel filtration chromatography (Superdex 75, GE Healthcare) in the same buffer conditions. *Hv* 1ALigN was purified using the same protocol but adding 2M KCl to all buffers.

### CD and Fluorescence Experiments

A JASCO J-810 spectropolarimeter with a quartz cuvette of 0.1 cm (1 cm) path length was used for the CD (fluorescence) experiments. For the CD experiments, samples were used at a concentration range of 2–4 µM and the thermal melts were run at 1 degree per minute, with monitoring at 222 nm with a bandwidth of 2 nm and a data pitch of 0.2 degrees. The thermal range of the experiment was optimized for every sample, assuring the proper determination of the baselines for both the folded and the unfolded states. Thermal denaturation curves monitored by fluorescence spectroscopy were collected at a concentration range of 1–2 µM, with measuring conditions equivalent to the CD experiments except for the use of a 2 nm excitation bandwidth centered at 280 nm. Between three and six independent measurements were obtained for each experimental condition from CD and fluorescence data, and duplicate points were used to obtain an estimation of the error. In all cases, the CD and fluorescence signal recovery after the thermal melt was monitored and is reported in the supplementary materials (Tables S1, S2, S3). When signal recovery was less than 95%, a duplicate was collected at a different scanning rate (3 deg/min). A variation of *T_m_* with the scanning rate is an indication of irreversible unfolding and mutants showing such change (within experimental error) were excluded from the analysis (red marks in Tables S1, S2, S3). Guanidinium chloride or urea denaturation experiments were followed by CD and used an initial volume of 1.7–2 mL at a protein concentration of 4 µM in 20 mM phosphate buffer at pH 6.0. Protein denaturation was achieved by the addition of aliquots of a solution at the same protein concentration but in 20 mM phosphate buffer and 6 M guanidinium chloride (GuHCl) or 10–11 M urea at pH 6.0, performed with an automatic titrator. GuHCl and urea concentrations were adjusted by measuring the refractive index. A comparison of the unfolding free energies obtained by chemical denaturation with guadinium chloride and urea are shown in Table S5. Due to low stability for ProtL Kx5E, Kx6E, and Kx7E, or limited solubility of NaCl and KCl in the denaturant conditions, chemical denaturation experiments were performed in the presence of different amounts of NaCl or KCl (0.25 M, 0.5 M, 0.75 M, 1 M, 1.5M, and 2.25M). The free energy showed a linear dependence with the NaCl or KCl concentration, and the intrinsic stability in the absence of salt for this ProtL mutant subset and the stability at 3.2 M salt were obtained by linear extrapolation.

To measure the fraction folded (FF) (at 25°C) in the presence of low amounts of KCl (Figure S2), CD spectra at 25°C and 85°C were recorded for each salt concentration. The FF has been calculated from the expression:
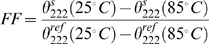
where 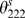 and 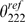 are the CD signal (ellipticity) at 222 nm for the sample at the given KCl concentration and for the reference sample (in 2M KCl), respectively.

Data analysis was completed with in-house built scripts programmed in Matlab (Simulink). Chemical and thermal denaturation data from CD and fluorescence spectroscopy were processed assuming the linear extrapolation method [24]: the molar ellipticity at each point of the transition can be described as a linear combination of the expected values for the folded (θ_F_) and unfolded (θ_U_) states. The values for θ_F_ and θ_U_ were obtained from extrapolations of the linear baselines. Detailed descriptions for the conversion of the experimental *T_m_* into Δ*G_salt_* and the calculation of *m_salt_* are given in the Protocol S1.

### NMR Measurements, Structure Elucidation, and Solvent Accessible Area Calculations

The ^1^H-^15^N-HSQC spectra for Kx7E were obtained from a single ^15^N-labeled 40 µM sample in 20 mM phosphate buffer at pH 6.0, in the absence and in the presence of 2 M NaCl. For Kx5Q/Kx6E, NMR experiments for the chemical shift assignment and collection of conformational restraints were performed on a single uniformly ^15^N- and ^13^C-labeled 300/200 µM protein sample dissolved in 93% H_2_O–7% D_2_O (v/v). NMR experiments were carried out at 300 K on a Bruker Avance 800 MHz spectrometer equipped with a cryoprobe. For Kx5Q/Kx6E, chemical shift assignments for 98%/97% of non-labile ^1^H, 97%/97% of ^15^N, and 94%/95% of ^13^C were obtained by using a combination of standard triple resonance experiments. Assignments were checked for consistency with 3D ^15^N-edited[^1^H,^1^H]-NOESY and ^13^C-edited[^1^H,^1^H]-NOESY. 3D NOESY spectra were recorded with 140 ms mixing time.

The 3D structures of the Kx5Q and Kx6E ProtL mutants were determined by combined automated NOESY cross peak assignment and structure calculations in torsion angle space with the software CYANA 2.1 [25]. The 20 conformers with the lowest final CYANA target function values were further refined by restrained energy minimization in a water shell with the program OPALp [26] using the AMBER force field [27]. MOLMOL [28] was used for the analysis and visualization of the structures. The 20 conformers that represent the solution structure of the Kx5Q and Kx6E mutants have been deposited in the Protein Data Bank with the accession codes 2jzp and 2kac, respectively.

The ensemble constituted by the 10 lowest energy conformations from the solution NMR structures of wild type ProtL (2ptl) [17] (without the his-tag tail) and the 10 lowest energy conformations for the Kx5Q ProtL (2jzp) and Kx6E ProtL (2kac) were employed for the solvent accessibility calculations, using the MOLMOL program [28] with a probe radius of 1.4 Å. Δ*ASA^WT−Mut^* for a Kx*n*Y mutant is determined from the expression:
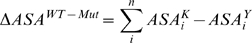
where for a given position i, 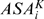, and 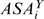 correspond to the solvent-exposed areas for wild type and mutated side chains, respectively, as determined from the high resolution structures. No experimental structural data are available for Q41, Q7, and E7. All the single K to Q and K to E mutants of ProtL were modeled using the Swiss Model Workspace [29] and the Protein Homology/analogY Recognition Engine (Phyre) [30]. Very similar results were found for the areas determined from the experimental and the modeled structures, and the average areas for Q41, Q7, and E7 determined from the modeled structures were used in the analysis. The same computational methods [29],[30] were used to build up homology models for the *Hv* and *Ec* 1ALigN proteins (WT for *Hv* 1ALigN and the appropriate mutant variants for *Hv* and *Ec* 1ALigN). For *Hv* 1ALigN, the structural models succeeded in creating a structure from residues 33 to 108 and this protein segment was used for the analysis of the including residues. For the excluded residues (D26, E26, D28, E28, D31, and E31), peptide models were used instead.

The ^15^N side-chain transversal relaxation rates were measured on the NHD isotopmer to circumvent the contribution from proton dipole-dipole cross-correlation [31]. Protein samples were lyophilized and resuspended in 50% D_2_O to maximize the population of the NHD isotopmer. Transversal relaxation spectra were recorded in a two-dimensional mode with relaxation times of 0, 5, 40 (×2), 80, 160, 200, and 240 ms. Experimental data were adjusted to exponential decays to obtain the relaxation constant (*T*_2_(^15^ *N*)).

### Solubility Measurements

The method used for the solubility measurements is very similar to the one described in [21]: an aliquot of 10 µL from concentrated protein stock (in 20 mM phosphate buffer, pH 6.0) was mixed with 10 µL of precipitating buffer (20 mM phosphate buffer, 4 M ammonium sulfate, pH6.0) in a 0.2 mL PCR tube. The mixtures were allowed to equilibrate for at least 1 min and were then transferred to 1.5 mL Eppendorf tubes and centrifuged for 2 min at 15,000 g. The concentrations of the supernatants were measured in a Nanodrop 1000 (Thermo Scientific). Four independent solubility measurements were carried out for each protein.

## Supporting Information

Figure S1 **Experimental raw data.** (A) The CD spectra (293 K) of wild type ProtL (red line), *Hv* 1ALigN (blue line), and *Ec* 1ALigN (green line) indicate that the three proteins are folded under the conditions of the study. (B–D) Temperature denaturation curves for some representative examples of ProtL (B and D) and *Hv* 1ALigN (C). Salt concentration is colour coded following the legend shown in each panel.
(0.37 MB TIF)

Figure S2 **Stability of wild type** ***Hv*** **1ALigN (25°C) at low concentrations of KCl.** Fraction of protein folded versus the concentration of KCl. The FF has been estimated from CD spectra, following a protocol described in the Materials and Methods. The error bars have been calculated from duplicate data. Buffer conditions: 20 mM phosphate buffer pH 8.0.
(0.07 MB TIF)

Figure S3 **Thermal denaturation** ***T_m_*** **values for ProtL.** Experimental mid denaturation points for the set of ProtL mutants as a function of the NaCl concentration. Error (black) bars represent the mean value for the duplicates. The lines represent the linear regressions for each specific dataset. At a given panel, the symbol and the colour identify the number of mutations incorporated: circle and 14% grey, square and 22% grey, diamond and 30% grey, leftward pointing triangle and 45% grey, rightward pointing triangle and 60% grey, pentagram and 75% grey, hexagram and 84% grey, or asterisk and 100% black correspond to 1, 2, 3, 4, 5, 6, 7, and 8 (or more) substitutions, respectively. Data for wild type ProtL are represented by blue circles and a blue line. All datasets are represented by filled symbols but the Kx*n*S mutants that are represented by open ones.
(0.27 MB TIF)

Figure S4 **Thermal denaturation** ***T_m_*** **values for** ***Hv*** **and** ***Ec*** **1ALigN.** Experimental mid denaturation points for wild type *Hv* 1ALigN, *Ec* 1ALigN, and the set of mutants as a function of the KCl concentration. Error bars represent the mean value for the duplicates. The lines represent the linear regressions for each specific dataset. At a given panel, the symbol and the colour identify the number of mutations incorporated: circle and 14% grey, square and 22% grey, diamond and 30% grey, leftward pointing triangle and 45% grey, rightward pointing triangle and 60% grey, pentagram and 75% grey, hexagram and 84% grey, or asterisk and 100% black correspond to 1, 2, 3, 4, 5, 6, 7, and 8 (or more) substitutions, respectively. Data for wild type *Hv* LigN (*Ec* LigN) are represented by red (green) circles (squares) and a red (green) line. All datasets are represented by filled symbols but the Sx*n*K mutants are represented by open ones.
(0.22 MB TIF)

Figure S5Correlation between the free energy of the mutants at 3.2 M salt (KCl or NaCl) determined by equilibrium denaturation experiments in urea (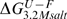 (experimental)) and the equivalent free energies estimated from the *m_salt_* values. Circles, squares, and diamonds correspond to ProtL, *Hv* 1ALigN, and *Ec* 1ALigN mutants, respectively. The expression: 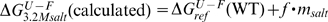 has been used to estimate the free energies at high salt concentration. Values used for 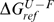 (WT) are: 4.5, 2.2, and 2.2 kcal·mol^−1^ for ProtL, *Hv* 1ALigN, and *Ec* 1ALigN, respectively, and the reference conditions are stated in Figure S5. Values used for the *f* factor are 3.2 for ProtL and *Ec* 1ALigN. Because the reference condition for *Hv* 1ALigN is at 1 M KCl, an *f* value of 2.2 was used for this protein instead.(0.10 MB TIF)

Figure S6 **Experimental** ***m_salt_*** **values for the cumulative mutants obtained from alternative mutation pathways.** The alternative mutation pathways are shown in red. The protein target and the mutation class are specified in the enclosed legend.
(0.22 MB TIF)

Figure S7 **Comparison of ^1^H-^15^N-HSQC spectra for wild type and Kx7E ProtL.** In the presence of 2 M NaCl, the signal dispersion in the ^1^H-^15^N-HSQC spectrum of wild type (A) and Kx7E (B) ProtL are very similar, indicating that the protein fold is preserved.
(0.15 MB TIF)

Protocol S1 **Calculation of the** ***m_salt_*** **slopes from the experimental** ***T_m_*** **values.**
(0.13 MB PDF)

Table S1 **Primers for the ProtL (multiple) mutants and degree of reversibility upon thermal unfolding.**
(0.02 MB PDF)

Table S2 **Primers for the** ***Hv*** **1ALigN (multiple) mutants and degree of reversibility upon thermal unfolding.**
(0.01 MB PDF)

Table S3 **Primers for the** ***Ec*** **1ALigN (multiple) mutants and degree of reversibility upon thermal unfolding.**
(0.01 MB PDF)

Table S4 **Summary of the experimental restraints and statistics of the structure determination of the ProtL Kx5Q and ProtL Kx6E proteins.**
(0.01 MB PDF)

Table S5 **Comparison of unfolding free energies (25E°C) obtained using guanidinium chloride (GuHCl) or urea.**
(0.01 MB PDF)

## Abbreviations

a.r.: acidic residues
CD: circular dichroism
*Ec* 1ALigN: 1A domain of the NAD^+^-dependent DNA ligase N from *Escherichia coli*
FF: fraction folded
GuHCl: guanidinium chloride
^1^H-^15^N-HSQC: heteronuclear single quantum correlation experiment
*Hv* 1ALigN: 1A domain of the NAD^+^-dependent DNA ligase N from *Haloferax volcanii*
IPTG: isopropyl-beta-D-thiogalactopyranoside
NMR: nuclear magnetic resonance spectroscopy
NOESY: nuclear overhauser effect spectroscopy
PCR: polymerase chain reaction
ProtL: IgG binding domain of the protein L from *Streptococcus magnus*
