# Supplementary figures and images for: Structural Basis for the Aminoacid Composition of Proteins from Halophilic Archea
Source: PLoS Biol. 2009 Dec 15;7(12):e1000257. doi: 10.1371/journal.pbio.1000257 (PMC2780699; doi:10.1371/journal.pbio.1000257)

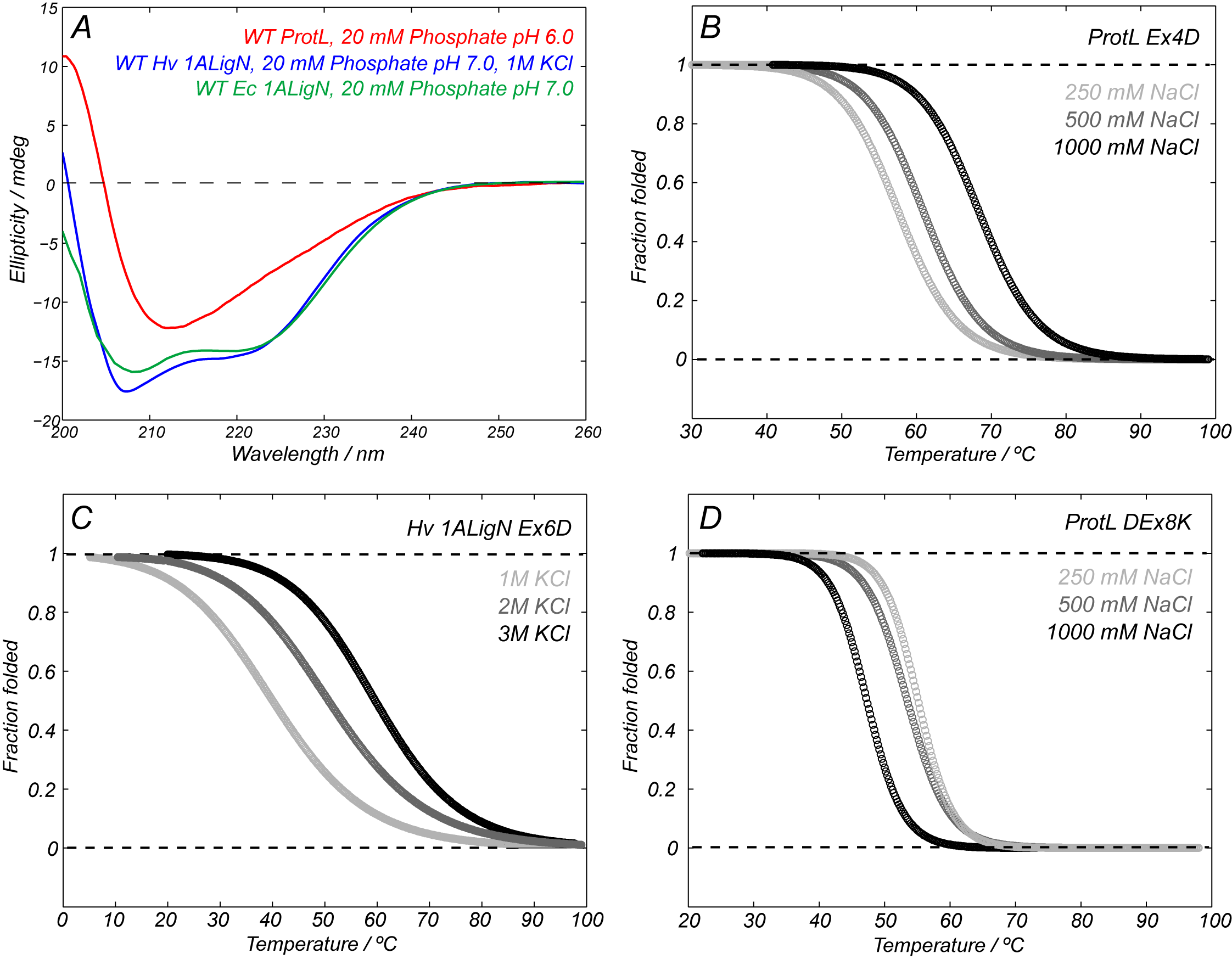

Supplement: Figure S1 — Experimental raw data. (A) The CD spectra (293 K) of wild type ProtL (red line), Hv 1ALigN (blue line), and Ec 1ALigN (green line) indicate that the three proteins are folded under the conditions of the study. (B–D) Temperature denaturation curves for some representative examples of ProtL (B and D) and Hv 1ALigN (C). Salt concentration is colour coded following the legend shown in each panel. (0.37 MB TIF) [file pbio.1000257.s001.tif]

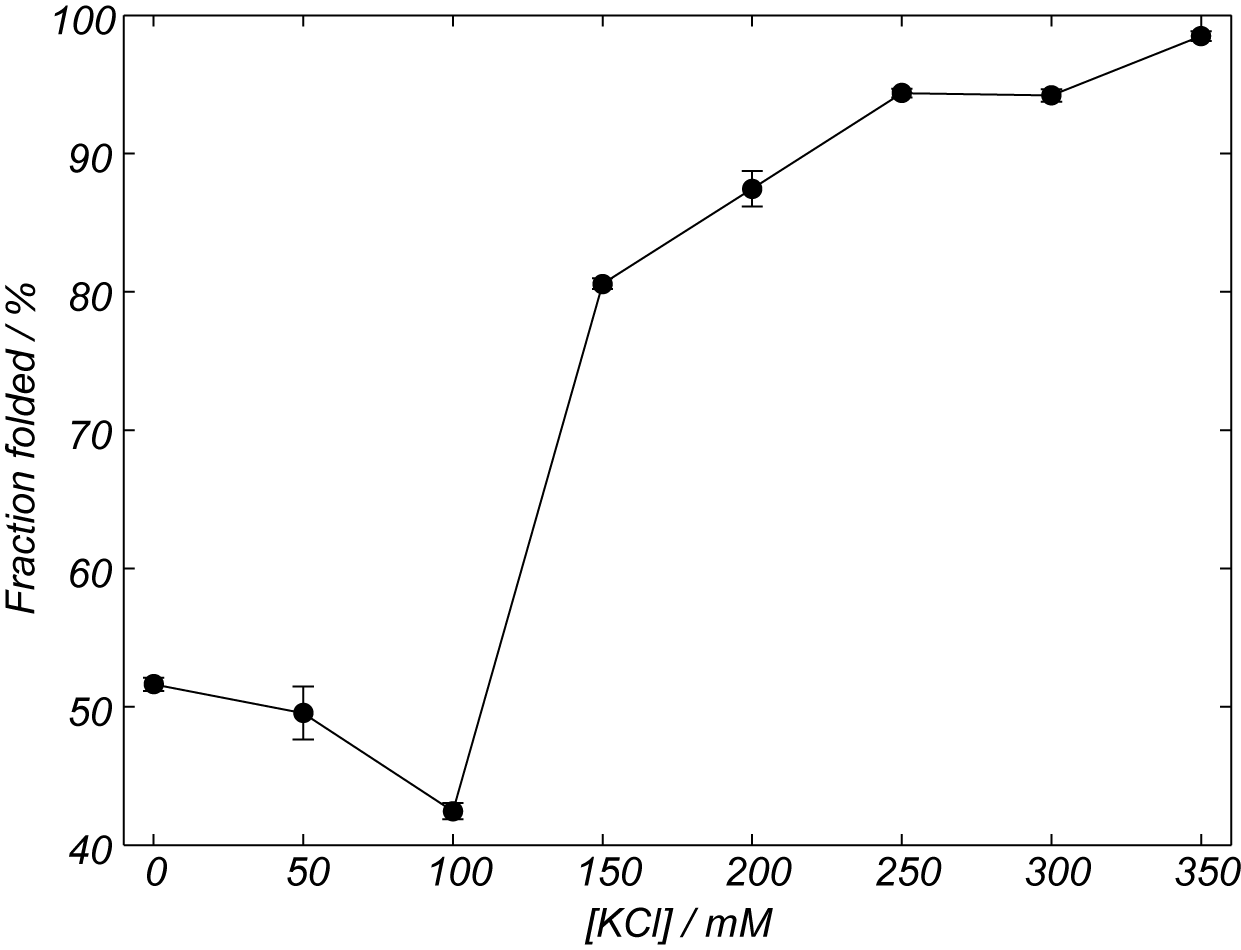

Supplement: Figure S2 — Stability of wild type Hv 1ALigN (25°C) at low concentrations of KCl. Fraction of protein folded versus the concentration of KCl. The FF has been estimated from CD spectra, following a protocol described in the Materials and Methods. The error bars have been calculated from duplicate data. Buffer conditions: 20 mM phosphate buffer pH 8.0. (0.07 MB TIF) [file pbio.1000257.s002.tif]

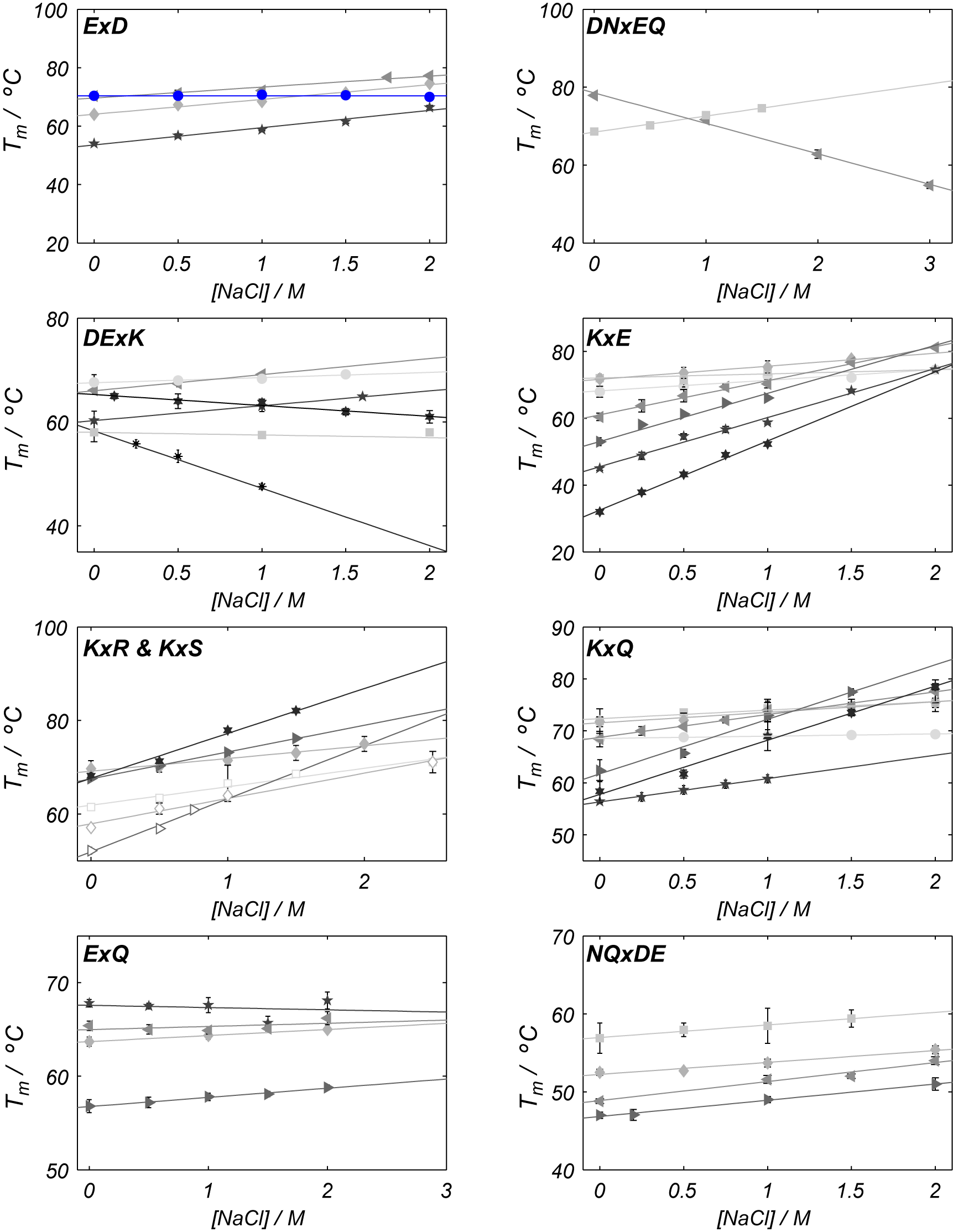

Supplement: Figure S3 — Thermal denaturation Tm values for ProtL. Experimental mid denaturation points for the set of ProtL mutants as a function of the NaCl concentration. Error (black) bars represent the mean value for the duplicates. The lines represent the linear regressions for each specific dataset. At a given panel, the symbol and the colour identify the number of mutations incorporated: circle and 14% grey, square and 22% grey, diamond and 30% grey, leftward pointing triangle and 45% grey, rightward pointing triangle and 60% grey, pentagram and 75% grey, hexagram and 84% grey, or asterisk and 100% black correspond to 1, 2, 3, 4, 5, 6, 7, and 8 (or more) substitutions, respectively. Data for wild type ProtL are represented by blue circles and a blue line. All datasets are represented by filled symbols but the KxnS mutants that are represented by open ones. (0.27 MB TIF) [file pbio.1000257.s003.tif]

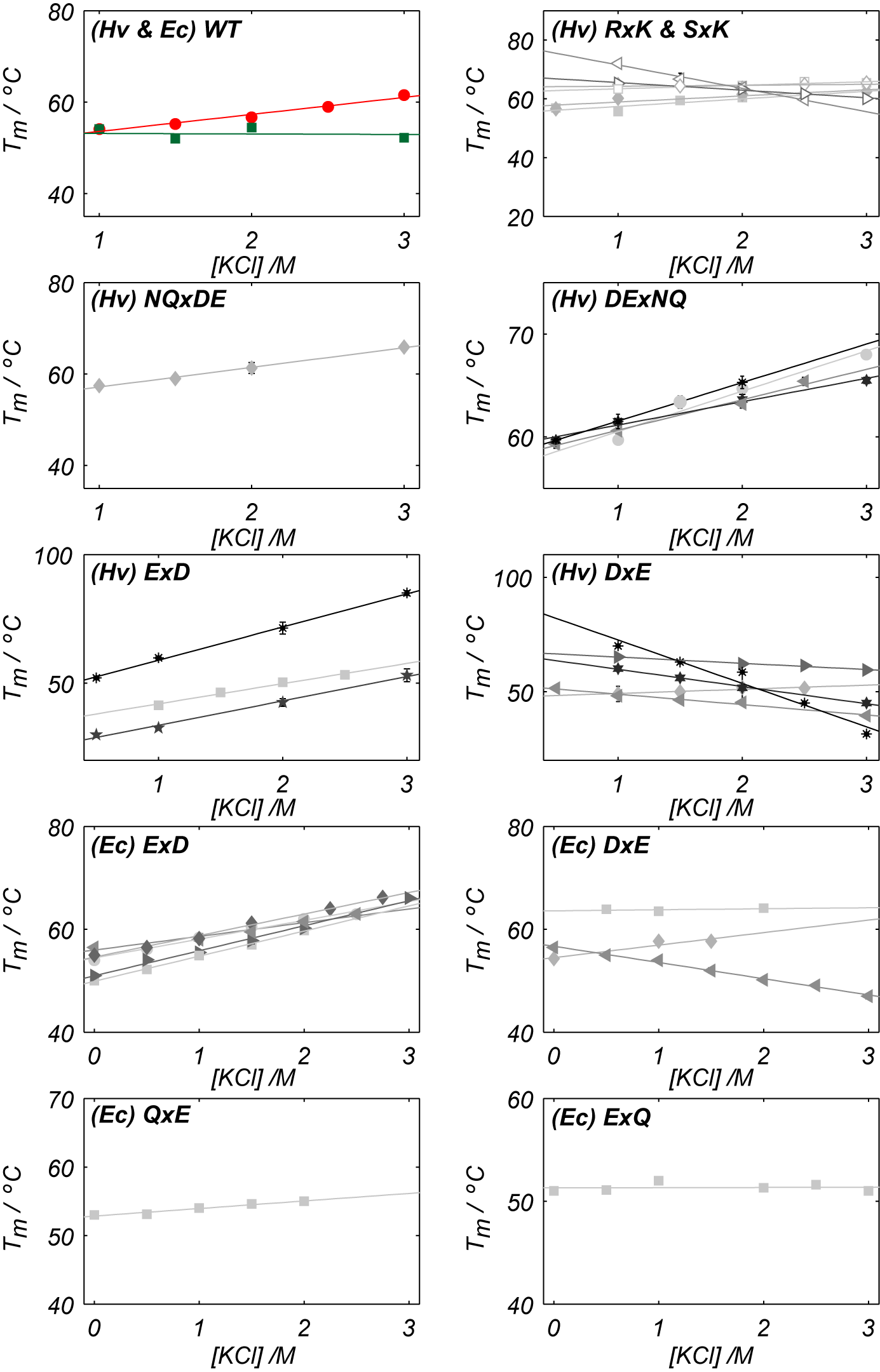

Supplement: Figure S4 — Thermal denaturation Tm values for Hv and Ec 1ALigN. Experimental mid denaturation points for wild type Hv 1ALigN, Ec 1ALigN, and the set of mutants as a function of the KCl concentration. Error bars represent the mean value for the duplicates. The lines represent the linear regressions for each specific dataset. At a given panel, the symbol and the colour identify the number of mutations incorporated: circle and 14% grey, square and 22% grey, diamond and 30% grey, leftward pointing triangle and 45% grey, rightward pointing triangle and 60% grey, pentagram and 75% grey, hexagram and 84% grey, or asterisk and 100% black correspond to 1, 2, 3, 4, 5, 6, 7, and 8 (or more) substitutions, respectively. Data for wild type Hv LigN (Ec LigN) are represented by red (green) circles (squares) and a red (green) line. All datasets are represented by filled symbols but the SxnK mutants are represented by open ones. (0.22 MB TIF) [file pbio.1000257.s004.tif]

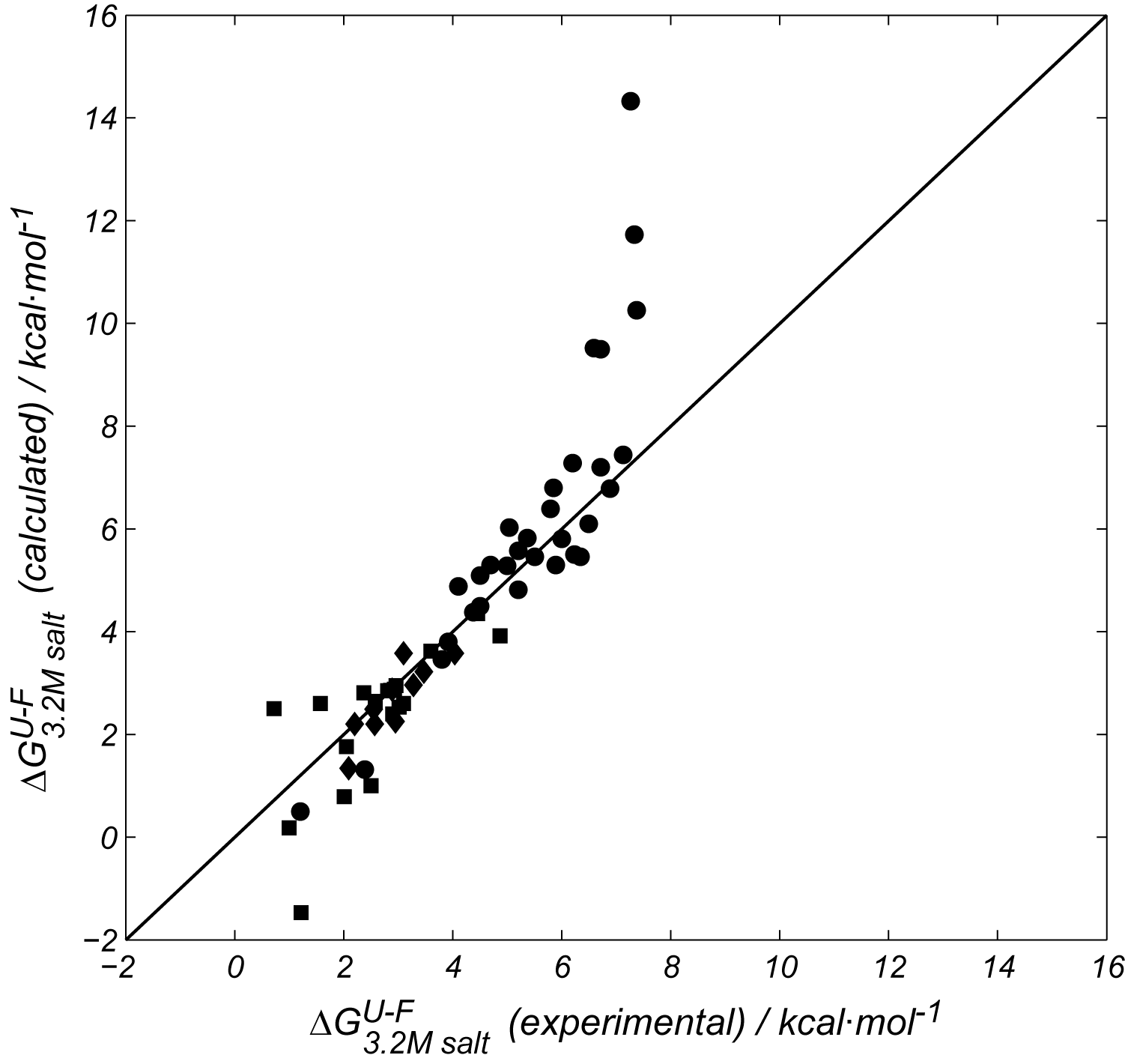

Supplement: Figure S5 — Correlation between the free energy of the mutants at 3.2 M salt (KCl or NaCl) determined by equilibrium denaturation experiments in urea ( (experimental)) and the equivalent free energies estimated from the msalt values. Circles, squares, and diamonds correspond to ProtL, Hv 1ALigN, and Ec 1ALigN mutants, respectively. The expression: has been used to estimate the free energies at high salt concentration. Values used for (WT) are: 4.5, 2.2, and 2.2 kcal·mol−1 for ProtL, Hv 1ALigN, and Ec 1ALigN, respectively, and the reference conditions are stated in Figure S5. Values used for the f factor are 3.2 for ProtL and Ec 1ALigN. Because the reference condition for Hv 1ALigN is at 1 M KCl, an f value of 2.2 was used for this protein instead. (0.10 MB TIF) [file pbio.1000257.s005.tif]

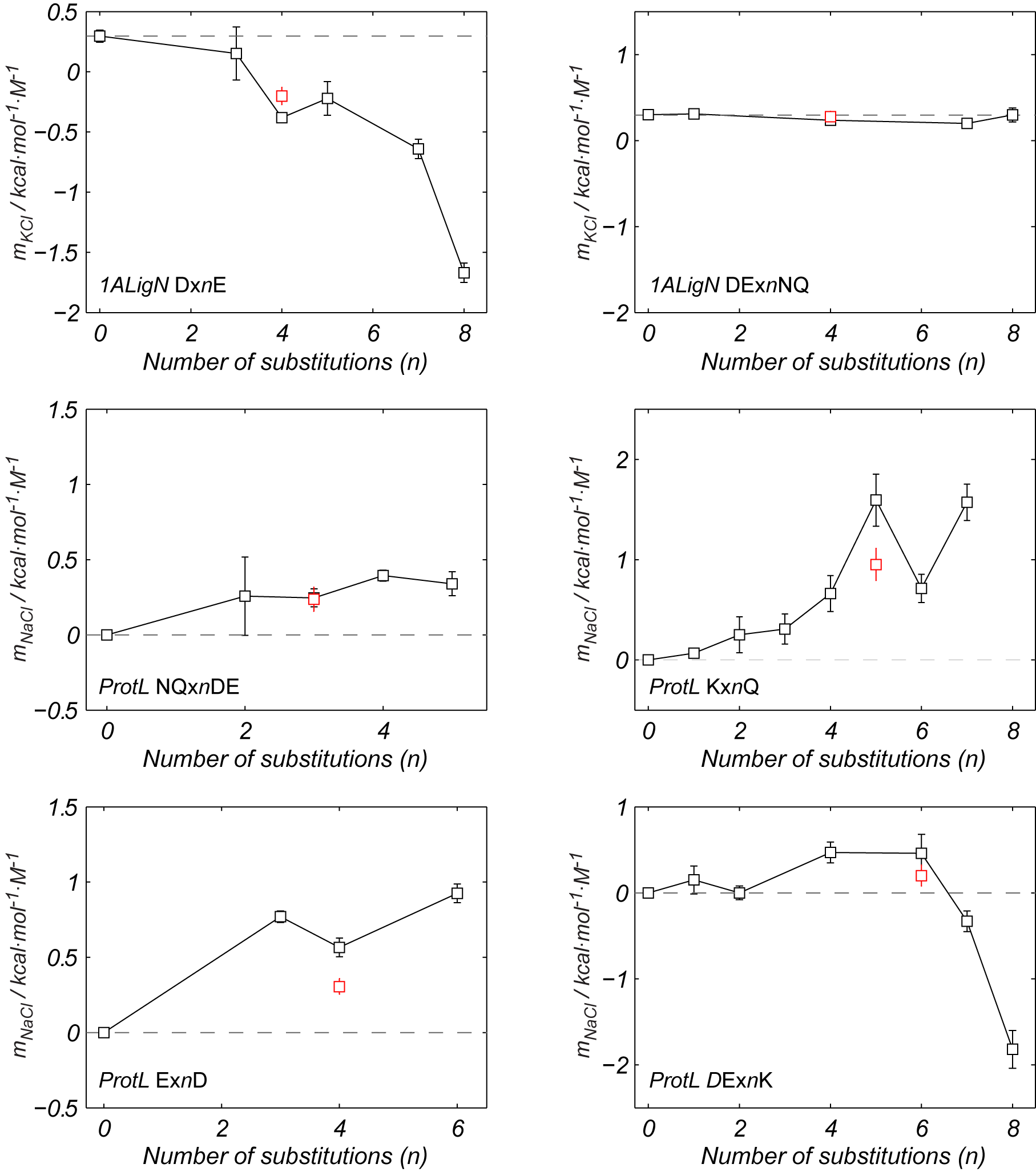

Supplement: Figure S6 — Experimental msalt values for the cumulative mutants obtained from alternative mutation pathways. The alternative mutation pathways are shown in red. The protein target and the mutation class are specified in the enclosed legend. (0.22 MB TIF) [file pbio.1000257.s006.tif]

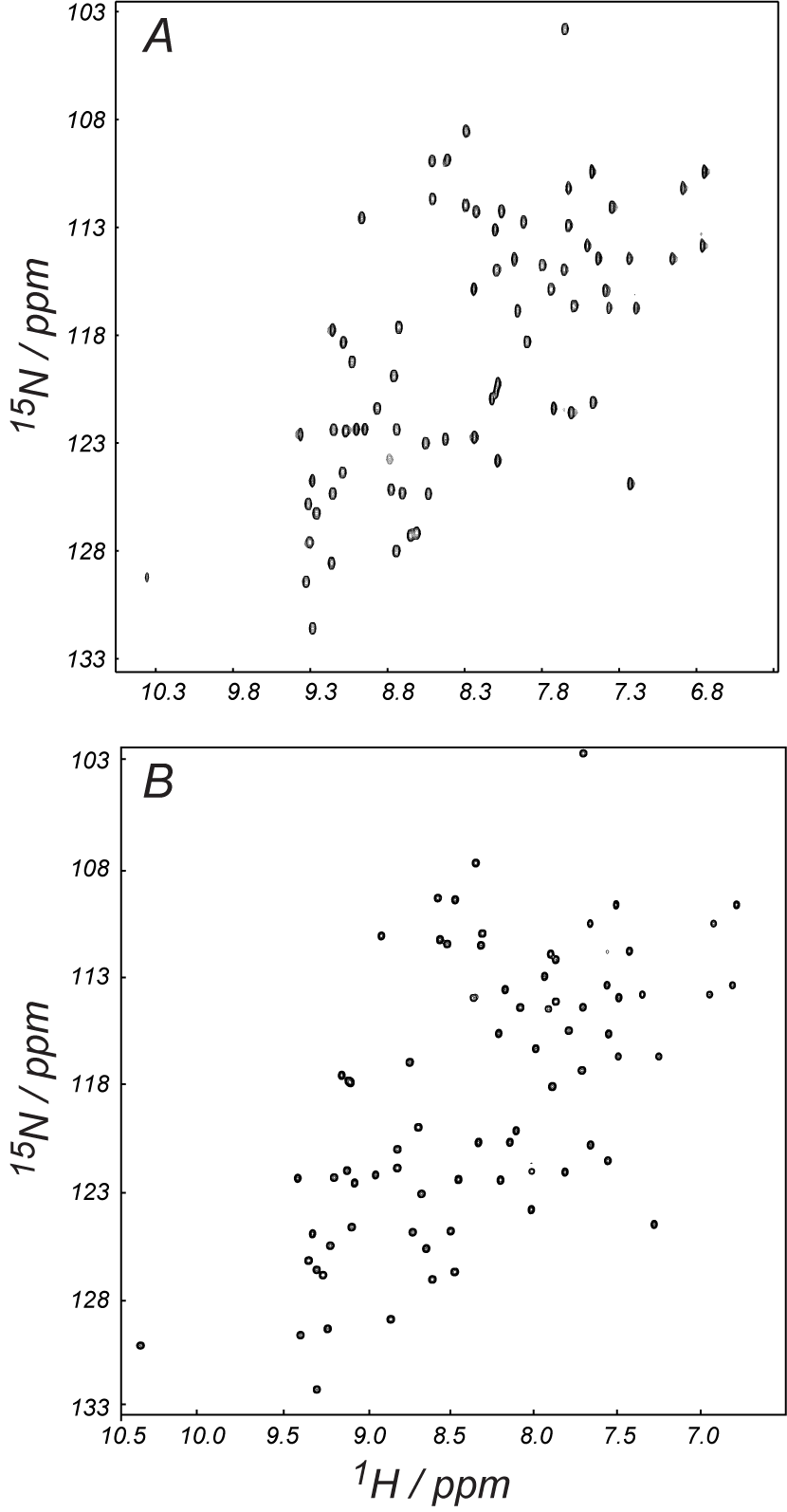

Supplement: Figure S7 — Comparison of 1H-15N-HSQC spectra for wild type and Kx7E ProtL. In the presence of 2 M NaCl, the signal dispersion in the 1H-15N-HSQC spectrum of wild type (A) and Kx7E (B) ProtL are very similar, indicating that the protein fold is preserved. (0.15 MB TIF) [file pbio.1000257.s007.tif]
