# Supplementary material for: Structural Basis for the Aminoacid Composition of Proteins from Halophilic Archea
Source: PLoS Biol. 2009 Dec 15;7(12):e1000257. doi: 10.1371/journal.pbio.1000257 (PMC2780699; doi:10.1371/journal.pbio.1000257)
Supplement: Protocol S1 — Calculation of the msalt slopes from the experimental Tm values. (0.13 MB PDF) [file pbio.1000257.s008.pdf]

PROTOCOL S1: CALCULATION OF THE m_{salt} SLOPES FROM THE EXPERIMENTAL T_m VALUES.

For the wild type forms of *Hv* 1ALigN, *Ec* 1ALigN and ProtL the curves of protein stability versus temperature have been estimated from equilibrium denaturation experiments (using guanidinium chloride) and thermal denaturation experiments (Figure A).

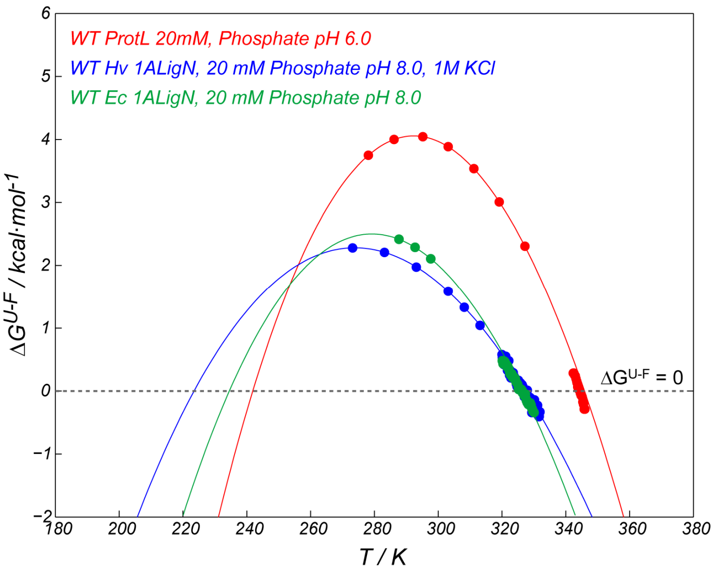

Figure A: Protein stability versus temperature for wild type ProtL (red), (previously reported in (1)), wild type *Hv* 1ALigN (blue) and wild type *Ec* 1ALigN (green). Circles correspond to the experimental $\Delta G^{U\text{-}F}$ points whereas solid lines show the best fitting to the Gibbs-Helmholtz equation.

The thermodynamic parameters have been determined by fitting the experimental data to the Gibbs-Helmholtz curve:

Table A: *Thermodynamic parameters for WT ProtL, WT Hv 1ALigN and WT Ec 1ALigN.*

Parameter	***WT ProtL***	***WT Hv 1ALigN***	***WT Ec 1ALigN***
ΔH_m / kcal·mol^{-1}	53.0 ± 4.8[(1,2)]	27.0 ± 2.5[(1)]	28.9 ± 2.1[(1)]
ΔC_p / kcal·mol^{-1}·K^{-1}	0.876 ± 0.05[(1)]	0.461 ± 0.02[(1)]	0.650 ± 0.1[(1)]

(1) The error has been estimated by Montecarlo propagation of the individual experimental errors, obtained from duplicates.

(2) Differential scanning calorimetry data for wild type ProtL gives a value of 54.5 kcal·mol^{-1}

Free energy changes upon salt addition can be calculated (for a given protein and with respect to a reference state) from the experimental T_m values (2):

$$\Delta G_{salt} = \Delta H_m^{salt}\left(1 - \frac{T_m^0}{T_m^{salt}}\right) - \Delta C_p^* \cdot \left[\left(T_m^{salt} - T_m^0\right) + T_m^0 \cdot \ln\left(\frac{T_m^0}{T_m^{salt}}\right)\right]$$

where T_m^{salt} and ΔH_m^{salt} are the melting temperature and melting enthalpy in the presence of salt respectively and ΔC_p^* is the apparent heat capacity change in the presence of cosolute. The label "*salt"* accounts for KCl or NaCl and T_m^0 is the melting temperature at the reference state (specified in Figure A for *Hv* 1ALigN, *Ec* 1ALigN and ProtL).

ΔC_p^* can be estimated from the slope of a plot of ΔH_m versus T_m (2). For each mutant and at every salt concentration, T_m and ΔH_m values have been obtained from the fitting of the thermal denaturation curves. Plots of ΔH_m versus T_m result in flat lines with negligible slopes. Figure B shows the plot of ΔH_m versus their respective T_m for all the mutants and conditions studied.

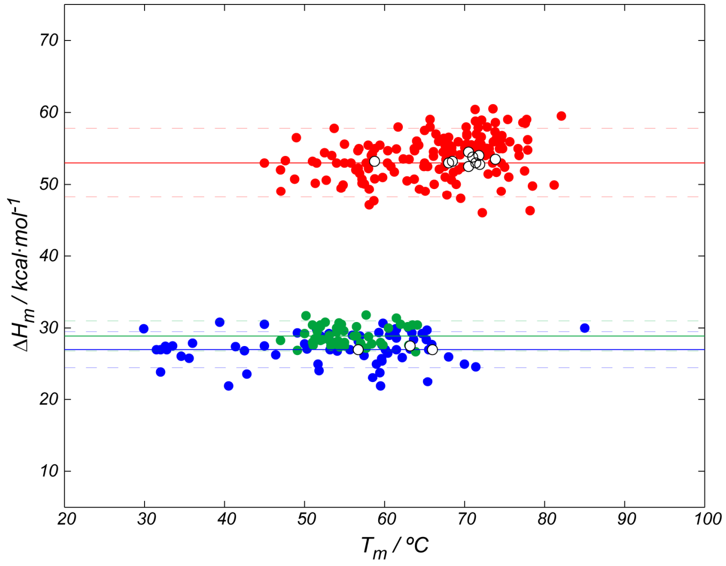

Figure B: ΔH_m versus T_m for the ProtL (red circles), *Hv* 1ALigN (blue circles) and *Ec* 1ALigN (green circles) datasets. All the mutants and each salt concentration tested in the present study are included in the plot. Values for wild type proteins (solid lines) and their confidence limits (dashed lines) are also represented. For a subset of data, ΔH_m have also been determined from DSC data (white circles).

ΔH_m values are always very close to the ones for the reference state (represented by solid lines in Figure B), almost always falling within the error limits for the reference state (shown as dashed lines in Figure B). For a selected set of data, the ΔH_m values have also been obtained from differential scanning calorimetry data (see below). The calorimetric ΔH_m values show less scattering from the reference enthalpies. All together it can be concluded that ΔC_p^*(ProtL) = ΔC_p^*(*Hv* 1ALigN) = ΔC_p^*(*Ec* 1ALigN) = 0 and the free energy changes upon salt addition have been determined from the equation:

$$\Delta G_{salt} = \Delta H_m^0 \left(1 - \frac{T_m^0}{T_m^{salt}} \right)$$

where ΔH_m^0 is equal to 53, 28.9 and 27 kcal·mol^{-1} for ProtL, *Ec* 1ALigN and *Hv* 1ALigN respectively and T_m^0 is the reference state for the three proteins (ProtL = 20 mM phosphate, pH 6.0; *Hv* 1ALigN = 20 mM phosphate, pH 8.0, 1M KCl; *Ec* 1ALigN = 20 mM phosphate, pH 8.0).

ΔG_{salt} varies linearly with respect to the molar salt concentration and the slope (m_{salt}) is used as a metric for haloadaptation:

$$m_{salt} = \left(\frac{\partial \Delta G_{salt}}{\partial C_m} \right)$$

where C_m is the molar salt concentration.

Estimation of the error. Error in m_{salt} has been propagated from the experimental uncertainties of the T_m by montecarlo analysis: for each T_m, 10000 random variants have been generated within the limits of the experimental uncertainty and they have been transformed into ΔG_{salt}. This dataset has been used to generate an ensemble of m_{salt} values. The standard deviation of this distribution is used as the error estimation for m_{salt}.

DSC data. For differential scanning calorimetry (DSC) measurements, sample concentration oscillated between 0.4 mM and 1.6 mM. For a given buffer condition, calorimeter was pre-equilibrated overnight and samples were run from 15 ºC to 95 ºC at 1 ºC/min. Data analysis included cubic baseline-progression and two state model data fitting with the apparatus-associated software based in Origin (MicroCal). Data shown in Figure B corresponds to ProtL: wild type (no salt, 500 mM NaCl and 1M NaCl), Kx1Q (no salt), Kx2Q

(no salt), Kx3Q (no salt and 1M NaCl), Kx5Q (2M NaCl), Kx5E (2M NaCl) and Ex6D (1M NaCl) and 1ALigN: wild type (2M KCl), Ex4Q (2M and 3M KCl) where "no salt" means means 20 mM phosphate buffer at pH 6.0.

1. Tadeo X, Pons M, Millet O (2007) Influence of the Hofmeister anions on protein stability as studied by thermal denaturation and chemical shift perturbation. *Biochemistry* 46: 917-923.
2. Santoro MM, Bolen DW (1988) Unfolding free energy changes determined by the linear extrapolation method. 1. Unfolding of phenylmethanesulfonyl alpha-chymotrypsin using different denaturants. *Biochemistry* 27: 8063-8068.
