# Supplementary material for: Structural Basis for the Aminoacid Composition of Proteins from Halophilic Archea
Source: PLoS Biol. 2009 Dec 15;7(12):e1000257. doi: 10.1371/journal.pbio.1000257 (PMC2780699; doi:10.1371/journal.pbio.1000257)
Supplement: Table S1 — Primers for the ProtL (multiple) mutants and degree of reversibility upon thermal unfolding. (0.02 MB PDF) [file pbio.1000257.s009.pdf]

Table S1: *Primers for the ProtL (multiple) mutants and degree of reversibility upon thermal unfolding.*

**Name**[(1)]	**Mutated residues**[(2)]	**Template**	**Forward primer**	**Analyzed**[(3)]	**Rev. / %**[(4)]
**Ex1D**	32	*WT*	5'-AAAAAGCAACTAGTGACGCTTATGCATATGCAGATAC-3'	*No*	-
**Ex2D**	32,46	*Ex1D*	5'-GAAAGACAATGGAGACTGGACTGTCGACG-3'	***Yes***	***85***
**Ex3D**	27,32,46	*Ex2D*	5'-GGAACATTTGACAAAGCAACATCAGACGCTTATGCG-3'	***Yes***	***94***
**Ex4D**	21,27,32,46	*Ex3D*	5'-CCACACAAACTGCAGACTTCAAAGGAAC-3'	*No*	-
Ex4D'	2,27,32,46	*EX3D*	5'-GGAGATATACCATGGATGAAGTAACAATCAAAGCTAACC-3'	***Yes***	***98***
**Ex5D**	3,21,27,32,46	*Ex4D*	5'-GGAGATATACCATGGAAGACGTAACAATCAAAGCTAACC-3'	*No*	-
**Ex6D**	2,3,21,27,32,46	*EX5D*	5'-GGAGATATACCATGGATGACGTAACAATCAAAGCTAACC-3'	***Yes***	***100***
**DNx1EQ**	38	*WT*	5'-GCATATGCAGAGACTTTGAAGAAAGACAATGG-3'	*No*	-
**DNx2EQ**	38,43	*DNx1EQ*	5'-GCAGAAACTTTGAAGAAAGAGAATGGAGAATGGACTGTAGATGTTGC-3'	***Yes***	***100***
**DNx3EQ**	38,43,44	*DNx2EQ*	5'-GCAGAGACTTTGAAGAAAGAGCAGGGAGAATGGACTGTAG-3'	***Yes***	***99***
**DNx4EQ**	38,43,50,53	*DNx2EQ*	5'-TGGACTGTAGAGGTTGCAGAGAAAGGTTATACTTTAAAT-3'	***Yes***	***89***
**DNx6EQ**	9,14,38,43,50,53	*DNx4EQ*	5'-GTAACAATCAAAGCTCAGCTAATCTTTGCACAGGGAAGCACACAA-3'	***Yes***	***50***
**DNx7EQ**	9,14,38,43,44,50,53	*DNx6EQ*	5'-GCAGAGACTTTGAAGAAAGAGCAGGGAGAATGGACTGTAG-3'	***Yes***	***40***
DNx7EQ'	9,14,38,43,50,53,59	*DNx6EQ*	5'-CCAGCAAATTTAATCTGTAAAGTATAACCTTTCTCTGCAAC-3'	***Yes***	***39***
**DNx8EQ**	9,14,38,43,44,50,53,59	*DNx7EQ*	5'-GTTGCAGAGAAAGGTTATACTTTACAGATTAAATTTGCTGG-3'	***Yes***	***41***
**NQx2DE**	9,14	*WT*	5'-GTAACAATCAAAGCTGACCTAATCTTTGCAGATGGAAGCACA-3'	***Yes***	***96***
**NQx3DE**	9,14,59	*NQx2DE*	5'-GATGTTGCAGATAAAGGTTATACTTTAGATATTAAATTTGCTGG-3'	***Yes***	***100***
NQx3DE'	9,14,44	*NQx2DE*	5'-CAGATACTTTGAAGAAAGACGATGGAGAATGGACTGT-3'	***Yes***	***94***
**NQx4DE**	9,14,44,59	*NQx3DE*	5'-GATGTTGCAGATAAAGGTTATACTTTAGATATTAAATTTGCTGG-3'	***Yes***	***100***
**NQx5DE**	9,14,18,44,59	*NQx4DE*	5'-CTAATCTTTGCAGATGGAAGCACAGAAACTGCAGAATTC-3'	***Yes***	***100***
**Ex1Q**	2	*WT*	5'-GGAGATATACCATGCAAGAAGTAACAATCAAAGCTAACC-3'	*No*	-
**Ex2Q**	2,27	*Ex1Q*	5'-GCAGAATTCAAAGGAACATTTGAACAGGCAACTAGTGAAGC-3'	*No*	-
**Ex3Q**	2,21,27	*Ex2Q*	5'-CCACACAAACTGCACAATTCAAAGGAAC-3'	***Yes***	***100***
**Ex4Q**	2,21,27,32	*Ex3Q*	5'-GCAACTAGTCAAGCTTATGCATATGCAGATAC-3'	*No*	-
**Ex5Q**	2,21,27,32,46	*Ex4Q*	5'-GAAAGACAATGGACAATGGAC-3'	***Yes***	***100***
**Ex6Q**	2,3,21,27,32,46	*Ex5Q*	5'-GGAGATATACCATGCAACAAGTAACAATCAAAGCTAACC-3'	***Yes***	***86***
**Kx1R**	7	*WT*	5'-GGAAGAAGTAACAATCAGAGCTAACCTAATCTTTGCAAATGG-3'	*No*	-
**Kx3R**	7,41,42	*Kx1R*	5'-TATGCGTATGCAGATACTTTGAGGAGAGACAATGGAG-3'	***Yes***	***95***
**Kx5R**	7,23,28,41,42	*Kx3R*	5'-CTGCAGAATTCAGAGGAACATTTGAAAGAGCAACATCAGAAG-3'	***Yes***	***100***
**Kx7R**	7,23,28,41,42,54,61	*Kx5R*	5'-TGTTGCAGATAGAGGTTATACTTTAAATATTAGATTTGCT-3'	***Yes***	***100***
**Kx1S**	7	*Kx1R*	5'-GGAAGAAGTAACAATCAGTGCTAACCTAATCTTTGCAAATGG-3'	***Yes***	***85***
**Kx1SKx2R**	7	*Kx3R*	5'-GGAAGAAGTAACAATCAGTGCTAACCTAATCTTTGCAAATGG-3'	*No*	-

**Kx3S**	7,41,42	*Kx1SKx2R*	5'-TATGCGTATGCAGATACTTTGAGTAGTGACAATGGAG-3'	***Yes***	***100***
**Kx2SKx3R**	54,61	*Kx5R*	5'-TGTTGCAGATAGTGGTTATACTTTAAATATTAGCTTTGCT-3'	*No*	-
**Kx4SKx1R**	41,42	*Kx2SKx3R*	5'-TATGCGTATGCAGATACTTTGAGTAGTGACAATGGAG-3'	*No*	-
**Kx5S**	7,41,42,54,61	*Kx4SKx1R*	5'-GGAAGAAGTAACAATCAGTGCTAACCTAATCTTTGCAAATGG-3'	***Yes***	***99***
**Kx1E**	42	*WT*	5'-GCATATGCAGATACTTTGAAGGAGGACAATGGAGAATGGAC-3'	***Yes***	***100***
**Kx2E**	42,54	*Kx1E*	5'-GGACTGTCGACGTTGCAGATGAGGGTTATAC-3'	***Yes***	***100***
**Kx3E**	28,42,54	*Kx2E*	5'-GCAGAATTCAAAGGAACATTTGAAGAAGCAACTAGTGAAGC-3'	***Yes***	***100***
**Kx4E**	28,42,54,61	*Kx3E*	5'-GGTTATACTTTAAATATTGAGTTTGCTGG-3'	***Yes***	***100***
**Kx5E**	23,28,42,54,61	*Kx4E*	5'-CCACACAAACTGCAGAATTCGAAGGAACATTTGAAGAAGC-3'	***Yes***	***100***
**Kx6E**	23,28,41,42,54,61	*Kx5E*	5'-GCATATGCAGATACTTTGGAGGAAGACAATGGAGAATGGAC-3'	***Yes***	***96***
**Kx7E**	7,23,28,41,42,54,61	*Kx6E*	5'-GGAAGAAGTAACAATCGAGGCTAACCTAATCTTTGCAAATGG-3'	***Yes***	***100***
**Kx1Q**	42	*WT*	5'-GCATATGCAGATACTTTGAAGCAGGACAATGGAGAATGGAC-3'	***Yes***	***100***
**Kx2Q**	42,54	*Kx1Q*	5'-GGACTGTCGACGTTGCAGATCAGGGTTATAC-3'	***Yes***	***89***
**Kx3Q**	28,42,54	*Kx2Q*	5'-GCAGAATTCAAAGGAACATTTGAACAGGCAACTAGTGAAGC-3'	***Yes***	***100***
**Kx4Q**	28,42,54,61	*Kx3Q*	5'-GGTTATACTTTAAATATTCAGTTTGCGG-3'	***Yes***	***99***
**Kx5Q**	23,28,42,54,61	*Kx4Q*	5'-CCACACAAACTGCAGAATTCCAGGGAACATTTGAACAAGC-3'	***Yes***	***100***
Kx5Q'	7,28,42,54,61	*Kx4Q*	5'-GGAAGAAGTAACAATCCAGGCTAACCTAATCTTTGCAAATGG-3'	***Yes***	***100***
**Kx6Q**	23,28,41,42,54,61	*Kx5Q*	5'-GCATATGCAGATACTTTGCAGCAGGACAATGGAGAATGGAC-3'	***Yes***	***100***
**Kx7Q**	7,23,28,41,42,54,61	*Kx6Q*	5'-GGAAGAAGTAACAATCCAGGCTAACCTAATCTTTGCAAATGG-3'	***Yes***	***100***
**DEx1K**	50	*WT*	5'-GAATGGACTGTCAAGGTTGCAGATAAAGG-3'	*No*	-
**DEx2K**	2,50	*DEx1K*	5'-GGAGATATACCATGAAGGAAGTAACAATCAAAGCTAACC-3'	***Yes***	***96***
**DEx3K**	2,27,50	*DEx2K*	5'-GCAGAATTCAAAGGAACATTTAAGAAAGCAACTAGTGAAGC-3'	*No*	-
**DEx4K**	2,27,43,50	*DEx3K*	5'-GCAGATACTTTGAAGAAAAAGAATGGAGAATGGAC-3'	***Yes***	***97***
**DEx5K**	2,21,27,43,50	*DEx4K*	5'-CCACACAAACTGCAAAGTTCAAAGGAAC-3'	*No*	-
**DEx6K**	2,21,27,43,50,53	*DEx5K*	5'-TGGAGAATGGACTGTAAAGGTTGCAAAGAAAGGTTATAC-3'	***Yes***	***98***
DEx6K'	2,21,27,43,46,50	*DEx5K*	5'-CTTTGAAGAAAAAGAATGGAAAATGGACTGTAAAGGTTGCAG-3'	***Yes***	***98***
**DEx7K**	2,3,21,27,43,50,53	*DEx6K*	5'-GGAGATATACCATGAAAAAAGTAACAATCAAAGCTAACC-3'	***Yes***	***91***
**DEx8K**	2,3,21,27,43,46,50,53	*DEx7K*	5'-CTTTGAAGAAAAAGAATGGAAAATGGACTGTAAAGGTTGCAG-3'	***Yes***	***98***

(1) XYx*n*WZ involves *n* substitutions from X or Y to W or Z respectively. The quotation mark indicates that an alternative cumulative pathway has been used.
(2) From the sequence: MEEVTIKANLIFANGSTQTAEFKGTFEKATSEAYAYADTLKKDNGEYTVDVADKGYTLNIKFAG.
(3) *Yes* = Protein expressed, purified and tested over NaCl. *No* = DNA used as an intermediate for cloning.
(4) Reversibility upon unfolding. The value corresponds to the lower value found for all the salt concentrations. Values in red indicate that a change in the T_m is observed after varying the scanning rate in the experiment and these values have not been used in the study.
