# Supplementary material for: Structural Basis for the Aminoacid Composition of Proteins from Halophilic Archea
Source: PLoS Biol. 2009 Dec 15;7(12):e1000257. doi: 10.1371/journal.pbio.1000257 (PMC2780699; doi:10.1371/journal.pbio.1000257)
Supplement: Table S2 — Primers for the Hv 1ALigN (multiple) mutants and degree of reversibility upon thermal unfolding. (0.01 MB PDF) [file pbio.1000257.s010.pdf]

Table S2: *Primers for the Hv 1ALigN (multiple) mutants and degree of reversibility upon thermal unfolding.*

Name[(1)]	Mutated residues[(2)]	Template	Forward primer	Analyzed[(3)]	Rev. / %[(4)]
**Ex2D**	104,107	*WT*	5'-ACGCGCTGGATACGGTCGATCACGTCTAGG-3'	***Yes***	***98***
**Ex4D**	47,51,104,107	*Ex2D*	5'-CACTCCTCCGCGATGCCGTCCGTGATCACGACCAC-3'	*No*	-
**Ex5D**	31,47,51,104,107	*Ex4D*	5'-AACCCGCCGATTCGCTCTCGCGCGA-3'	*No*	-
**Ex6D**	26,28,47,51,104,107	*Ex4D*	5'-TTCCCTCCGACCGATTTCGATCCCGCCGA-3'	***Yes***	***100***
**Ex7D**	26,28,31,47,51,104,107	*Ex6D*	5'-AACCCGCCGATTCGCTCTCGCGCGA-3'	*No*	-
**Ex8D**	26,28,31,47,51,80,104,107	*Ex7D*	5'-GACTCGTTGCGCTCGATGACGCGTTC-3'	*No*	-
**Ex9D**	26,28,31,47,51,80,98,104,107	*Ex8D*	5'-TCGGCGGCGATCCCATCGACG-3'	***Yes***	***89***
**Dx1E**	101	*WT*	5'-GGCGAGCCCATCGAAGCGCTGGAGACGGT-3'	*No*	-
**Dx2E**	53,101	*Dx1E*	5'-GCCGTCCGTGAGCACGAACACCGCTACTAC G-3'	*No*	-
**Dx3E**	84,86,101	*Dx1E*	5'-CGCTCGAAGACGCGTTCGAACTCGAAACGACCAACA -3'	***Yes***	***95***
**Dx4E**	53,84,86,101	*Dx3E*	5'-GCCGTCCGTGAGCACGAACACCGCTACTACG-3'	***Yes***	***85***
Dx4E'	81,84,86,101	*Dx3E*	5'-GACTCGTCGCGCTCGAAGAAGCGTTCGAACTCGAAACGA -3'	***Yes***	***98***
**Dx5E**	53,81,84,86,101	*Dx4Ealt*	5'-GCCGTCCGTGAGCACGAACACCGCTACTACG-3'	*No*	-
**Dx7E**	53,66,70,81,84,86,101	*Dx5E*	5'-GCTCGTCTCCGAAGCGGCATACGAAGCGCTGTTCT -3'	***Yes***	***94***
**Dx8E**	53,61,66,70,81,84,86,101	*Dx7E*	5'-TCGCAGCCGAACCGCTCGTCTCCGA-3'	***Yes***	***90***
**NQx1DE**	41	*WT*	5'-CCGAAGGGGAGGCCGCACTCCTCCGCGAGGC-3'	*No*	-
**NQx2DE**	89,93	*WT*	5'-TCGACACGACCGACAGCCCGACGGACCGCGTCGG-3'	*No*	-
**NQx3DE**	41,89,93	*NQx2DE*	5'-CCGAAGGGGAGGCCGCACTCCTCCGCGAGGC-3'	***Yes***	***100***
**DEx1NQ**	12	*WT*	5'-CATCACCATCACCTTCAATCAACAAGTTTGTACAA-3'	***Yes***	***100***
**DEx3NQ**	26,28,31	*WT*	5'-CCCTCCGACCCAGTTCCAACCCGCCCAGTCGCTCTCG -3'	*No*	-
**DEx4NQ**	12,26,28,31	*DEx3NQ*	5'-CATCACCATCACCTTCAATCAACAAGTTTGTACAA-3'	***Yes***	***100***
DEx4NQ'	12,47,51,53	*DEx1NQ*	5'-CACTCCTCCGCCAGGCCGTCCGTCAGCACAACCACCGCTA-3'	***Yes***	***100***
**DEx7NQ**	12,26,28,31,47,51,53	*DEx4NQ*	5'-CACTCCTCCGCCAGGCCGTCCGTCAGCACAACCACCGCTA-3'	***Yes***	***92***
**DEx8NQ**	12,26,28,31,98,101,104,107	*DEx4NQ*	5'-TCGGCGGCCAGCCCATCAACGCGCTGCAGACGGTCCAACACG-3'	***Yes***	***100***
**Rx1K**	75	*WT*	5'-GCTGTTCTCCAAACTCGTCGCGCTCGAA-3'	***Yes***	***98***
**Rx2K**	35,75	*Rx1K*	5'-GAGTCGCTCTCGAAGGAGGCGGCCGAAG-3'	***Yes***	***97***
**Rx3K**	35,55,75	*Rx2K*	5'-GAGCACGACCACAAGTACTACGTCGCGG-3'	***Yes***	***99***
**Rx4K**	35,46,50,75	*Rx2K*	5'-GCACTCCTCAAGGAGGCCGTCAAGGAGCACGACC-3'	***Yes***	***100***
**Sx2K**	13,15	*WT*	5'-CACCATCACCTTGAAAAAACAAAGTTGTACAAAAAAGCAGGCTT-3'	***Yes***	-
**Sx3K**	13,15,74	*Sx2K*	5'-ACGACGCGCTGTTCAAACGACTCGTCGCGCTCGAAGACG-3'	***Yes***	-
**Sx4K**	13,15,32,34	*Sx2K*	5'-TCGAACCCGCCGAGAAGCTCAAGCGCGAGGCGGCCGAAG-3'	***Yes***	-
**Sx5K**	13,15,32,34,74	*Sx4K*	5'-ACGACGCGCTGTTCAAACGACTCGTCGCGCTCGAAGACG-3'	***Yes***	-

(1) XYx*n*WZ involves *n* substitutions from X or Y to W or Z respectively. The quotation mark indicates that an alternative cumulative pathway has been used. (2) From the sequence: MSYYHHHHHHLESTSLYKKAGFPPTEFEPAESLSREAAEGQAALLREAVREHDHRYYVAADPLVSDAAYDALFSRLVALEDAFDLDTTNSPTNRVGGE

PIDALETVEHV. (3) *Yes* = Protein expressed, purified and tested over KCl. *No* = DNA used as an intermediate for cloning. (4) Reversibility upon unfolding. The value corresponds to the lower value found for all the salt concentrations. Values in red indicate that a change in the T_m is observed after varying the scanning rate in the experiment and these values have not been used in the study.
