# Supplementary material for: Structural Basis for the Aminoacid Composition of Proteins from Halophilic Archea
Source: PLoS Biol. 2009 Dec 15;7(12):e1000257. doi: 10.1371/journal.pbio.1000257 (PMC2780699; doi:10.1371/journal.pbio.1000257)
Supplement: Table S3 — Primers for the Ec 1ALigN (multiple) mutants and degree of reversibility upon thermal unfolding. (0.01 MB PDF) [file pbio.1000257.s011.pdf]

Table S3: *Primers for the Ec 1ALigN (multiple) mutants and degree of reversibility upon thermal unfolding.*

Name(1)	Mutated residues(2)	Template	Forward primer	Analyzed(3)	Rev. / %(4)
**Ex1D**	41	*WT*	5'- ACGCTTCGCCATCATGATTATCTTTATCATGTGAT -3'	***Yes***	***100***
**Ex2D**	24,27	*WT*	5'-GCAGGCTTCATGGATTCAATCGATCAACAACTGACAGAACT-3'	***Yes***	***100***
**Ex3D**	63,66,68	*WT*	5'-TGATGCGCGATCTGCGCGATCTGGATACCAAACATCC-3'	***Yes***	***100***
**Ex4D**	41,63,66,68	*Ex1D*	5'- TGATGCGCGATCTGCGCGATCTGGATACCAAACATCC -3'	***Yes***	***100***
**Ex5D**	24,27,63,66,68	*Ex2D*	5'- TGATGCGCGATCTGCGCGATCTGGATACCAAACATCC -3'	***Yes***	***100***
**Dx2E**	54,58	*WT*	5'- AAATTCCCGAAGCTGAATACGAAAGGCTGATGCGCGAACT -3'	***Yes***	***100***
**Dx3E**	54,58,78	*Dx2E*	5'- AGAACTGATTACGCCTGAATCGCCTACCCAACGTGTA -3'	***Yes***	***100***
**Dx4E**	48,54,58,78	*Dx3E*	5'- CTTTATCATGTGATGGAAGCGCCGGAAATTCCCGA -3'	***Yes***	***100***
**Ex2Q**	24,27	*WT*	5'- GCAGGCTTCATGCAATCAATCCAACAACAACTGACAGAACT -3'	***Yes***	***100***
**Qx2E**	28,29	*WT*	5'- ATGGAATCAATCGAAGAAGAACTGACAGAACTGCGAACGA -3'	***Yes***	***100***

(1) XYx*n*WZ involves *n* substitutions from X or Y to W or Z respectively. The quotation mark indicates that an alternative cumulative pathway has been used. (2) From the sequence: MSYYHHHHHHLESTSLYKKAGFMESIEQQLTELRTTLRHHEYLYHVMDAPEIPDAEYDRLMRELRELETKHPELITPDSPTQRVGAAPLAAF (3) *Yes* = Protein expressed, purified and tested over KCl. *No* = DNA used as an intermediate for cloning. (4) Reversibility upon unfolding. The value corresponds to the lower value found for all the salt concentrations. Values in red indicate that a change in the T_m is observed after varying the scanning rate in the experiment and these values have not been used in the study.
