# Supplementary material for: Structural Basis for the Aminoacid Composition of Proteins from Halophilic Archea
Source: PLoS Biol. 2009 Dec 15;7(12):e1000257. doi: 10.1371/journal.pbio.1000257 (PMC2780699; doi:10.1371/journal.pbio.1000257)
Supplement: Table S4 — Summary of the experimental restraints and statistics of the structure determination of the ProtL Kx5Q and ProtL Kx6E proteins. (0.01 MB PDF) [file pbio.1000257.s012.pdf]

Table S5: *Summary of the experimental restraints and statistics of the structure determination of the ProtL Kx5Q and ProtL Kx6E proteins.*

Protein	***ProtL Kx5Q***	***ProtL Kx6E***
NOE upper distance limits:	1355	1438
■ *Short-range,* $|i - j| \leq 1$	631	641
■ *Medium-range,* $1 < |i - j| < 5$	226	243
■ *Long-range,* $|i - j| \geq 5$	498	554
ϕ/ψ dihedral angle restraints from TALOS	76	78
Maximal violation (Å)	0.14	0.13
Violations > 0.2 Å	0	0
CYANA target function ($Å^2$)	0.54 ± .11	0.45 ± 0.16
AMBER energy (kcal/mol)	3051.70 ± 55.66	1383.33 ± 46.57
Ramachandran plot statistics (%). Residues in:		
■ *most favoured regions*	88.4	89.9
■ *additionally allowed regions*	11.1	8.9
■ *generously allowed regions*	0.6	1.2
■ *disallowed regions*	0	0
RMSD to mean coordinates (Å)(1) backbone/heavy atoms (from 4 to 64)	0.34 / 0.70	0.39 / 0.74
RMSD to wild type ProtL, PDB ID 1hz6 (Å) (1)		
■ *backbone (from 4 to 64)*	0.77	0.99
■ *backbone (secondary structure)*	0.58	0.69

(1) Values over the 20 energy-refined conformers.
