# Supplementary material for: Structural Basis for the Aminoacid Composition of Proteins from Halophilic Archea
Source: PLoS Biol. 2009 Dec 15;7(12):e1000257. doi: 10.1371/journal.pbio.1000257 (PMC2780699; doi:10.1371/journal.pbio.1000257)
Supplement: Table S5 — Comparison of unfolding free energies (25E°C) obtained using guanidinium chloride (GuHCl) or urea. (0.01 MB PDF) [file pbio.1000257.s013.pdf]

Table S6: *Comparison of unfolding free energies (25ºC) obtained using guanidinium chloride (GuHCl) or urea.*

Protein	$\Delta G^{U\text{-}F}$***(GuHCl)***	$\Delta G^{U\text{-}F}$***(urea)***
Wild type ProtL	4.40 ± 0.1	4.30 ± 0.3
Kx1Q ProtL	4.40 ± 0.2	4.30 ± 0.4
Kx2Q ProtL	4.60 ± 0.3	4.70 ± 0.7
Kx3Q ProtL	4.55 ± 0.2	4.50 ± 0.2
Kx4Q ProtL	4.40 ± 0.1	4.50 ± 0.8
Kx5Q ProtL	3.65 ± 0.1	3.30 ± 0.4
Kx6Q ProtL	4.15 ± 0.2	4.00 ± 0.2
Kx7Q ProtL	3.50 ± 0.2	3.30 ± 0.3
Kx1E ProtL	4.50 ± 0.3	4.50 ± 0.2
Kx2E ProtL	4.45 ± 0.5	4.20 ± 0.3
Kx3E ProtL	4.50 ± 0.6	4.40 ± 0.1
Kx4E ProtL	3.80 ± 0.2	4.10 ± 0.2
Kx5E ProtL	0.30 ± 0.2	1.20 ± 0.2
Kx6E ProtL	0.50 ± 0.2	0.60 ± 0.1
Kx7E ProtL	-0.40 ± 0.2	-0.50 ± 0.4
Wild type *Hv* 1ALigN	1.80 ± 0.3	1.70 ± 0.2
